# The Nasal–Brain Drug Delivery Route: Mechanisms and Applications to Central Nervous System Diseases

**DOI:** 10.1002/mco2.70213

**Published:** 2025-06-06

**Authors:** Yi Qiu, Shiyuan Huang, Li Peng, Li Yang, Guosong Zhang, Tao Liu, Fang Yan, Xi Peng

**Affiliations:** ^1^ Sichuan Industrial Institute of Antibiotics School of Pharmacy Chengdu University Chengdu China; ^2^ Department of Endocrinology and Metabolism Shanghai Pudong Gongli Hospital Pudong New Area Shanghai China; ^3^ Department of Technology Sichuan Youngster Technology Co., Ltd Chengdu China; ^4^ Jiangxi University of Traditional Chinese Medicine Nanchang China; ^5^ Department of Geriatrics Geriatric Diseases Institute of Chengdu Chengdu Fifth People's Hospital Chengdu China

**Keywords:** blood–brain barrier, central nervous system diseases, drug delivery systems, intranasal administration, nose–brain drug delivery

## Abstract

The blood–brain barrier (BBB) is a highly selective and protective barrier that restricts the entry of most therapeutic agents into the central nervous system (CNS), posing a significant challenge for the treatment of CNS diseases. The nose‐to‐brain drug delivery (NBDD) route has emerged as a promising strategy to bypass the BBB, offering direct, noninvasive, and efficient transport of drugs to the brain. This review begins with a concise overview of the BBB structure and its biofunctions, followed by an in‐depth discussion of the mechanisms underlying the nose‐to‐brain pathway, including the olfactory and trigeminal nerve routes, and respiratory pathway. We further highlight the therapeutic research development of neurodegenerative diseases, acute neurological diseases, brain tumor, and psychiatric disorders when using NBDD drugs encompassing small‐molecule drugs, proteins, peptides, nucleic acids, siRNA, and herbal compounds, in which we also introduce innovative delivery systems, including nanocarriers and novel platforms such as exosomes, which enhance drug stability, targeting efficiency, and bioavailability. In addition, we provide a comprehensive overview of recent clinical advancements in therapeutics delivered via the intranasal route for CNS diseases. Finally, we discuss the challenges and future directions of NBDD, emphasizing its potential to transform the treatment landscape for CNS disorders.

## Graphical abstract

Τhe BBB restricts CNS drug delivery. NBDD bypasses the BBB via olfactory/trigeminal routes, enabling CNS delivery of therapeutic (small molecules, biologics, nucleic acids) for neurodegenerative/psychiatric disorders and brain tumors using advanced NBDD delivery systems (e.g nanocarriers) to enhance targeting and bioavailability.

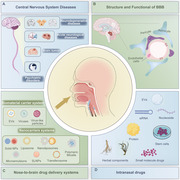

## 1 Introduction

The aging population's rapid growth in recent decades has led to a yearly increase in central nervous system (CNS) diseases, such as neurodegenerative disorders, acute neurological conditions, brain tumors, and depression [1, 2, 3]. Neurodegenerative diseases encompass Alzheimer's, Parkinson's, Huntington's, among others. Acute neurological diseases encompass conditions such as stroke and traumatic brain injury (TBI). These diseases exhibit pathological features including neurodegeneration, neuronal cell loss, excessive glial cell inflammation, and impaired blood–brain barrier (BBB) function [4, 5] Despite the introduction of new drugs, treatment efficacy is significantly limited by the complex mechanisms of CNS disorders, drug tolerance, and inadequate drug targeting [6]. Therefore, further improved therapy strategy are required in clinical management of CNS disorder.

Nose‐to‐brain drug delivery (NBDD) is an innovative method, with intranasal (IN) administration offering a reliable and convenient way to deliver therapeutics directly to the brain [7]. Nose‐to‐brain delivery offers significant benefits such as noninvasiveness, rapid action, CNS specificity, minimal side effects, and ease of self‐administration due to the nasal vasculature network and high permeability of nasal epithelia, bypassing first‐pass metabolism [8, 9]. One of the most important advantages of nose‐to‐brain delivery is the fast onset of action, unlike other routes of administration [10, 11]. This is mainly because of the direct transport of the drug through olfactory and trigeminal nerve pathways bypassing the BBB [12, 13]. On the other hand, this method has been utilized for delivering small‐molecule drugs, biopharmaceuticals, protein drugs, and therapies like stem cell and gene therapy [14, 15]. For example, IN administration of stem cells are used to treat diseases like multiple sclerosis (MS), psychiatric disorders, and neurodegenerative disorders like Alzheimer's disease (AD) [16, 17]. Moreover, nanotechnology‐based IN delivery such as nanoparticles (NPs), liposomes, and micelles are explored to improve the stability, solubility, and bioavailability of drugs in NBDD. To date, the therapeutic potential of NBDD drugs has been widely investigated in various neurological disease models. Inspiringly, some clinical drugs, such as Nayzilam, Spravato, and Valtoco, are all NBDD formulations, which have been approved by United States Food and Drug Administration (US FDA) and achieved good therapeutic effects in the treatment of CNS diseases [18, 19]. Furthermore, an NBDD form of insulin is undergoing pilot clinical trials as a potential treatment for AD [20].

In this review, we comprehensively summarize the structure of the BBB, the three pathways of nasal‐to‐brain delivery, drug delivery systems, and the therapeutic effects of some NBDD potential drugs including proteins, peptides, genes, stem cells, small molecule drugs, and herbal ingredients in the treatment of CNS diseases. We also discuss the current challenges in NBDD application and suggest future directions in this field.

## 2 Previous Development of NBDD Systems

### 2.1 The Structure of the BBB

The BBB is crucial for brain protection, regulating material transport and maintaining a stable neuronal environment [21, 22]. Unlike peripheral capillaries, the BBB features tightly connected endothelial cells, active efflux transporters, and major facilitator superfamily domain‐containing protein 2A (MFSD2A) [23, 24]. Transmembrane tight junction proteins (TJs), such as claudins, occludin, and junctional adhesion molecules, link to the intracellular actin cytoskeleton via proteins like zonula occludens‐1 [25]. These TJs inhibit paracellular diffusion, creating a physical barrier with low permeability, while MFSD2A regulates transcytosis, reinforcing the transcellular barrier [26, 27]. Endothelial cells, pericytes, and astrocytes form neurovascular units, interacting through secreted factors to regulate BBB microcirculation development and function [28]. There are two ways by which the BBB strictly regulates the transfer of various substances from blood to brain: through the paracellular pathway and transcellular pathway [29, 30, 31]. The paracellular pathway allows ions and solutes to passively diffuse based on concentration gradients [32, 33]. The transcellular pathway includes passive diffusion, absorptive transcytosis, receptor‐mediated transcytosis (RMT), and carrier‐mediated transport (CMT) [34]. Passive diffusion permits only small molecules under 400 Da or those with high lipid solubility [35, 36]. These transport mechanisms maintain synaptic function, information processing, and neuronal connections by regulating the brain's interstitial fluid composition, thus protecting the brain. Consequently, the BBB restricts entry of neurotoxic plasma components, blood cells, pathogens, and most drug molecules [37, 38].

**FIGURE 1:**
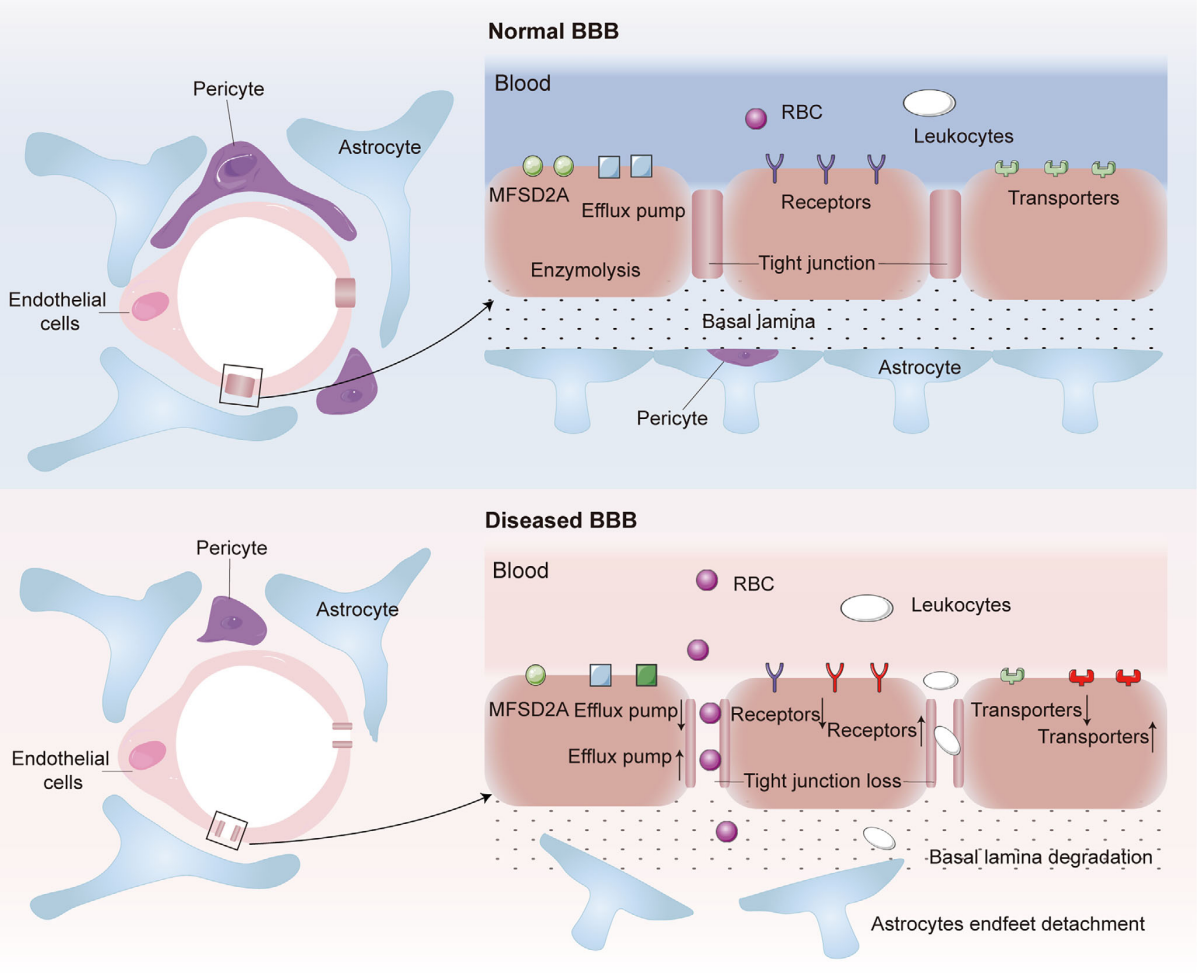
Schematic representation of the blood–brain barrier (BBB) in normal brain and the diseased BBB under pathological conditions. The BBB is composed of endothelial cells connected by tight junctions, supported by pericytes and astrocytes. In neurological diseases, the BBB undergoes structural and functional alterations, including increased permeability due to tight junction loss, enhanced transcytosis from reduced MFSD2A and/or increased caveolin‐1 expression, and impaired carrier‐mediated transport and receptor‐mediated transport functions. Additionally, cellular infiltration and degeneration of pericytes and endothelial cells contribute to BBB disruption. MFSD2A: major facilitator superfamily domain‐containing protein 2a.

BBB dysfunctions play a critical role in brain and CNS diseases, characterized by increased permeability from TJ loss and enhanced transcytosis due to MFSD2A deficiency or elevated caveolin‐1, impacting CMT and RMT functions [39, 40, 41]. For instance, during ischemic stroke (IS), the BBB becomes excessively permeable to macromolecules [42]. However, the degree and timing of BBB disruption in each disease are not well understood due to various limitations. For instance, both transient or chronic BBB permeability loss are commonly consistently observed in MS [43]. In the case of AD, early degeneration of cerebral microvasculature, reductions in cerebral blood flow, and subsequent breakdown of the BBB can exacerbate cerebrovascular degeneration, neuropathological changes, and cognitive impairment associated with AD [44]. In pathological conditions associated with TBI, the damage extends beyond mere physical harm to the blood vessels; hemorrhage and edema further exacerbate the disruption of the BBB at the lesion site and in surrounding areas [45, 46]. Given the complexity of CNS pathologies, current studies on BBB disruption across various diseases remain rudimentary. It is important to acknowledge that drug delivery through a compromised BBB presents significantly greater challenges than through an intact BBB. Alterations in the BBB can affect the accumulation and intracranial distribution of drugs by either facilitating or impeding their passage, thereby complicating the development and research of therapeutic agents. Consequently, understanding the key proteins and structures involved in the formation and regulation of the BBB is essential for advancing IN drug delivery strategies for a range of diseases. Furthermore, the administration of drugs via various nanocarriers represents a promising therapeutic approach for addressing BBB injury and dysfunction [47] (Figure 1).

### 2.2 The Pathways and Mechanisms for Nose‐to‐Brain Delivery

**FIGURE 2:**
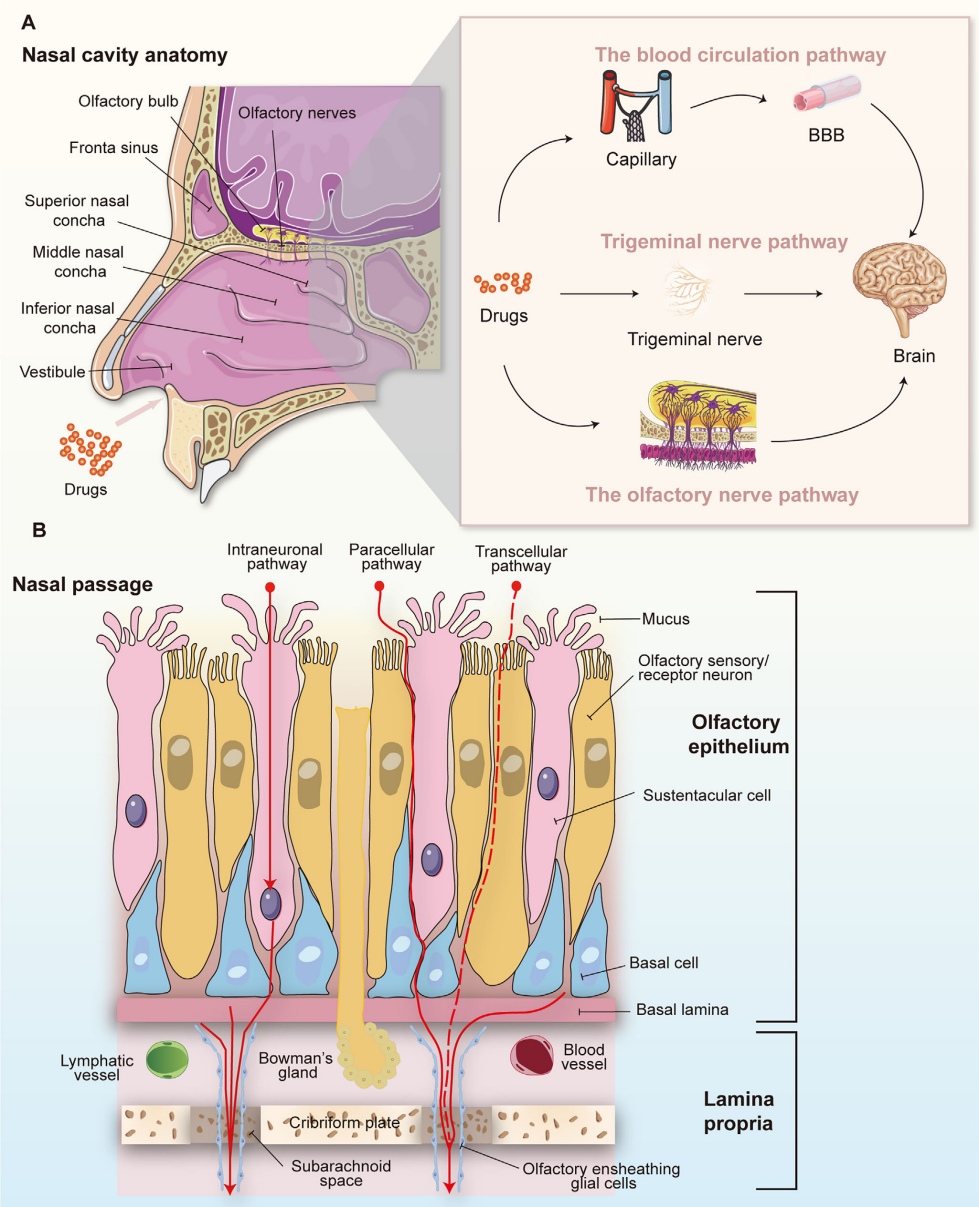
The pathways and mechanisms for nose‐to‐brain delivery. (A) Schematic diagram of the internal structure of nasal cavity and three routes of nasal brain administration. After nasal administration, some drugs undergo mucociliary clearance and enzymatic degradation in the nasal mucosa before reaching systemic and central nervous system (CNS) compartments. Drugs in the respiratory region near the inferior turbinate are absorbed into the bloodstream via the respiratory pathway. The nose‐to‐brain pathway primarily involves the olfactory and trigeminal nerve routes, as well as the respiratory pathway, which includes axonal transport through olfactory and trigeminal nerves. (B) The major components of the olfactory epithelium and lamina propria, along with the uptake routes from the nasal cavity. The olfactory region comprises the olfactory epithelium (OE) and lamina propria. The OE is surrounded by globose and horizontal basal cells and includes sustentacular cells, Bowman's glands (which produce mucus), and olfactory sensory neurons. The lamina propria, separated from the OE by a basal lamina, contains blood and lymphatic vessels. Drug transport across the epithelium can occur via paracellular or transcellular pathway, allowing molecules to pass through tight junctions or cells to reach the lamina propria. From there, drugs can enter systemic circulation via blood or lymphatic vessels. Additionally, olfactory sensory neurons provide a direct intracellular pathway for brain delivery.

The nose is essential for breathing and smelling, with its nasal cavity consisting of two narrow chambers about 12–14 cm long and lined with a mucosal layer [48]. Its total surface area is roughly 150 cm^2^. Each nasal cavity is divided into three regions: vestibule, respiratory, and olfactory [49]. The vestibule, the most anterior part, has a small surface area (approximately 0.6 cm^2^) and limited vasculature, leading to minimal drug absorption [50]. In contrast, the respiratory region has the largest surface area and abundant vasculature, with innervation by the trigeminal nerve's ophthalmic and maxillary branches, providing sensory input to the nasal cavity [51, 52]. Trigeminal nerve neurons are located in the semilunar ganglion, with axons reaching the brainstem via the pons [53]. Lateral branches traverse the cribriform plate to the olfactory bulb, forming a pathway for drug access to the brain [54]. However, the trigeminal nerve terminals beneath the mucosal epithelium are not conducive to drug transport [55]. The respiratory region's mucosa, with its rich blood flow and extensive surface area, is crucial for systemic drug delivery [56]. The olfactory region, in the upper nasal cavity, has a smaller mucosal area but is significant for IN drug delivery [57]. Cells in the olfactory epithelium include neurons, supporting cells, basal cells, and Bowman's gland cells as shown in Figure 2 [58]. The olfactory bulb connects to the piriform cortex, amygdala, and hypothalamus, enabling direct drug delivery to the brain [59, 60]. Overall, there are three pathways that contribute to the NBDD, namely, olfactory nerve, trigeminal nerve, and respiratory pathway.

The principal route for the transport of drug molecules into the brain is through the olfactory and trigeminal pathways, which facilitate the distribution of these drugs to various brain regions, ensuring their distribution across the entire organ [61, 62]. Drug molecules are primarily conveyed via two mechanisms: intraneuronal transport primarily involves the internalization of olfactory or trigeminal neurons via nonspecific processes or receptor‐mediated endocytosis [63]. Once inside, drug molecules are encapsulated in vesicles and transported along the axon to nerve endings, where they are released into the synaptic cleft and bind to postsynaptic cells in the olfactory bulb, eventually reaching various brain regions [64, 65]. Additionally, the extra‐neuronal pathway, which includes paracellular and transcellular transport, is a significant mechanism for IN delivery [66].

In the paracellular transport pathway, drug molecules move through the TJs between nasal epithelial cells to access the lamina propria [49]. TJs do not influence the permeability of lipophilic molecules, but hydrophilic molecules must traverse this barrier [67]. This pathway is optimal for molecules with a molecular weight under 1 KDa, with bioavailability diminishing as molecular weight increases. Olfactory neurons have a lifespan of about 30−60 days, during which they are not fully replaced [59]. This allows the opening of tight TJs between cells, enabling molecules, including macromolecules, to enter the brain [68]. Transcellular pathways, effective for lipophilic drugs, primarily occur in the nasal epithelium's supporting cells and involve mechanisms like receptor‐mediated endocytosis, passive diffusion, or liquid‐phase endocytosis [69, 70]. Additionally, certain excipients in nasal formulations can expand TJs and enhance drug translocation [71]. Typically, intraneural transport takes hours for the molecule to reach various brain regions, whereas extra‐neural pathways deliver drugs directly to the brain within minutes [72].

### 2.3 Drug Delivery Systems

While the IN route offers advantages, it also presents limitations, including limited absorption time, the necessity for small doses (25−200 µL) due to restricted surface area, rapid mucociliary clearance, and enzymatic degradation. In addition, the physical and chemical properties of drugs, such as molecular size, solubility, and lipophilicity, can influence their absorption in the nasal cavity and accumulation in the brain [7].

To enhance therapeutic outcomes of drugs, a variety of delivery strategy have been employed in CNS disease research. Nanocarriers, viral vectors, EVs carriers, and intelligent responsive drug delivery systems developed in recent years can effectively encapsulate drugs, ensure sustained drug release, increase drug stability, and improve drug penetration [73].

Currently, viral vectors can be used for gene delivery to patients with neurological disorders [74]. However, their application is limited by manufacturing challenges, high production costs, and significant safety concerns, including patient fatalities in clinical trials [75]. Despite these issues, viral vectors remain a prospective area for future research. Similarly, exosomes have been used to transport small molecules, proteins, and nucleic acids across the BBB [76]. For example, an exosome linked to RAGE‐binding‐peptide was developed as a hypoxia‐specific carrier for NBDD of anti‐microRNA oligonucleotide [77].

Traditionally, nanocarrier systems have been pivotal in advancing drug delivery technologies over recent decades [78, 79, 80]. Various nanocarriers, such as lipid nanoparticulate systems, liposomes, nanoemulsions, microemulsions, polymeric micelles, dendrimers, transfersomes, and nanosuspensions, have been effectively utilized to enhance CNS drug delivery via IN [81, 82, 83, 84]. These carriers offer significant benefits, including improved drug solubility and increased bioavailability through enhanced absorption. They can incorporate ligands for targeted organ delivery and are considered biodegradable and biocompatible carriers [85, 86]. A key strategy for brain targeting involves surface‐modified nanocarriers functionalized with ligands like proteins, peptides, and monoclonal antibodies [87, 88]. For example, RVG29 peptide‐modified polyethylene glycol‐polylactic acid‐co‐glycolic acid NPs possess physicochemical properties exhibit stable and sustained drug release properties, with demonstrated brain‐targeting efficacy in a cerebral ischemia (CI) rat model [89]. Another approach to enhance absorption and bioavailability is the use of mucoadhesive nasal formulations, which increase retention at the application site [83, 84]. For example, quercetin has been formulated into a mucoadhesive nanoemulsion capable of crossing the BBB and preferentially accumulating in the brain, thereby reducing systemic side effects [90]. Nonetheless, there has been development in the field of intelligent responsive drug delivery systems, such as the thermosensitive hydrogel system (THS), have demonstrated promising smart response and controlled release properties. In a study conducted by Zhou et al. [91], THS‐loaded resveratrol was employed as a model drug, exhibiting characteristics such as slow release, temperature sensitivity, cytocompatibility, and a significant increase in neurotransmitter levels following low‐dose IN administration.

The potential of these delivery systems for clinical drug formulation depends on completing quality evaluations, process optimization, and preclinical studies, including toxicological assessments of excipients, permeation enhancers, and polymers [92]. The advancement of novel drug delivery systems has significantly broadened the therapeutic applications of drugs for CNS disorder treatment.

## 3 IN Delivery of Drugs for CNS Treatment

Recent evidence indicates that IN administration plays a significant role in treating CNS disorders, particularly neurodegenerative diseases like Alzheimer's and Parkinson's. This method facilitates the effective delivery of large‐molecule therapeutics to the brain, thereby enhancing therapeutic efficacy. Various chemical drugs and biological agents, including peptides, proteins, and RNA, have been developed for IN delivery. This part reviews recent research on IN drug administration for treating CNS disorders.

Encouraged by the promising therapeutic potential of IN administration in animal studies for CNS diseases, several clinical trials have been launched to evaluate this delivery method's feasibility. Currently, the Clinical Trial database (www.clinicaltrials.gov) lists about 150 clinical trials examining the safety, distribution, and therapeutic outcomes of IN drugs, including completed, ongoing, and planned studies. These trials will be systematically organized in Table 1.

**TABLE 1:** Summary of the application of intranasal drugs in clinical trials for the treatment of CNS diseases.

Disease	Phase	Drug	Study name	State	NCT number
AD	Phase 2	Insulin	SNIFF 120: Study of nasal insulin to fight forgetfulness (120 days) (2006)	Completed	NCT00438568
	Phase 2	Insulin glulisine	Safety and effectiveness study of intranasal insulin glulisine on cognitive and memory in mild‐mod AD patients (2011)	Completed	NCT01436045
	Phase 1 and 2	AD‐SVF Cells	Study to assess the safety and effects of autologous adipose‐derived stromal cells in patients with Alzheimer's disease (2015)	Withdrawn	NCT02912169
	Phase 2	Insulin with semaglutide	Combination of intranasal insulin with oral semaglutide to improve cognition and cerebral blood flow: a feasibility study (2024)	Recruiting	NCT06072963
	Phase 2	Insulin and empagliflozin	Study of nasal insulin to fight forgetfulness—combination intranasal insulin and empagliflozin trial (2021)	Completed	NCT05081219
	Phase 2	Insulin by Aptar Pharma CPS intranasal delivery device	Study of nasal insulin to fight forgetfulness (SNIFF)—3‐week Aptar CPS device (2025)	Not yet recruiting	NCT05006599
	Not Applicable	BMSC	Neurologic bone marrow derived stem cell treatment study (2016)	Recruiting	NCT02795052
PD	Phase 1	Glutathione	Central nervous system uptake of intranasal glutathione in Parkinson's disease (2014)	Completed	NCT02324426
	Phase 2	Glutathione	Phase IIb study of intranasal glutathione in Parkinson's disease (2015)	Completed	NCT02424708
	Phase 1	glutathione	A phase 1 study of intranasal reduced glutathione in Parkinson's disease (2012)	Completed	NCT01398748
	Phase 2	POD l‐dopa	Therapeutic potential for intranasal levodopa in Parkinson's disease‐off reversal (2018)	Completed	NCT03541356
	Phase 2	Insulin	Single center safety and tolerability trial of intranasal insulin in Parkinson's disease (2020)	Active, not recruiting	NCT04251585
	Phase 2	Insulin	Evaluating the effect of intranasal insulin administration on motor and non‐motor symptoms in Parkinson's disease patients; a randomized double‐blinded placebo‐controlled clinical trial (2020)	Completed	NCT04687878
	Phase 1	hFGF‐1	A clinical research study evaluating the safety, tolerability and efficacy of intranasally administered human FGF‐1 in subjects with Parkinson's disease (2022)	Unknown status	NCT05493462
	Phase 1	CYR‐064 theophylline nasal spray	A single‐arm study to evaluate the feasibility of CYR‐064 theophylline nasal spray for the treatment of Parkinson's disease related hyposmia and anosmia (2025)	Not yet recruiting	NCT06498687
MS	Phase 1	Dexamethasone	Nasal administration of dexamethasone for multiple sclerosis (MS) treatment(2008)	Withdrawn	NCT00674141
	Phase 1	Foralumab	A phase 1b, Double‐blind, randomized, placebo controlled, multiple ascending dose study of the safety, tolerability and immune effects of the intranasal anti‐CD3 monoclonal antibody foralumab in primary and secondary progressive MS(2021)	Withdrawn	NCT05029609
	Phase 2	Methylprednisolone	Towards the improvement of the treatment of acute relapses in multiple sclerosis: a randomized double‐blind, non‐inferiority controlled trial comparing intranasal versus intravenous methylprednisolone (2023)	Enrolling by invitation	NCT06223074
	Phase 1and 2	Insulin	Intranasal insulin for improving cognitive function in multiple sclerosis (2017)	Completed	NCT02988401
		BMSC	Neurologic bone marrow derived stem cell treatment study (2016)	Not Applicable	NCT02795052
ALS	Phase 1 and 2	hUC‐MSC‐sEV‐001	An exploratory study on the use of intranasal administration of small extracellular vesicles for the treatment of amyotrophic lateral sclerosis (2024)	Recruiting	NCT06598202
	Not applicable	BMSC	Neurologic bone marrow derived stem cell treatment study (2016)	Recruiting	NCT02795052
Stroke	Phase 2	Insulin	Intranasal insulin and post‐stroke cognition: a pilot study (2016)	Completed	NCT02810392
	Phase 4	Progesterone	An international multicenter, randomized, double‐blind, placebo‐controlled clinical trial of progesterone combined intranasal and intramuscular administration in patients with acute hemorrhagic stroke (2020)	Unknown status	NCT04143880
	Phase 4	NGF	Effects of intranasal nerve growth factor for acute ischemic stroke (2016)	Completed	NCT03686163
	Phase 1 and 2	Conditioned medium with umbilical cord MSCs	Safety and efficacy of combined conditioned medium with umbilical cord mesenchymal stem cells as a novel strategy for acute stroke infarct (2022)	Unknown status	NCT05008588
	Phase 1 and 2	MSCs	Adult mesenchymal stromal cells to regenerate the neonatal brain: the PASSIoN trial (perinatal arterial stroke treated with stromal cells intranasally) (2020)	Completed	NCT03356821
	Phase 1	E‐selectin	Induction of mucosal tolerance to human E‐selectin for the secondary prevention of stroke (2003)	Withdrawn	NCT00069069
TBI	Observational	Dexmedetomidine	Intranasal dexmedetomidine sedation for pediatric computerized tomography imaging (2013)	Unknown status	NCT01900405
	Phase 1and 2	Bioactive factors produced by autologous M2 macrophages	Safety/efficacy of intranasally‐administered bioactive factors produced by autologous M2 macrophages in patients with organic brain syndrome (2016)	Completed	NCT02957123
Glioblastoma	Phase 1	temozolomide	Pilot study to evaluate the safety, tolerability and effectiveness of intranasal administration of temozolomide in patients with glioblastoma (2019)	Completed	NCT04091503
	Phase 1 and 2	NEO100 (perillyl alcohol)	An open‐label, phase 1/2A dose escalation study of safety and efficacy of NEO100 in recurrent or progressive grade III or grade IV gliomas with IDH1 Mutation (2017)	Recruiting	NCT02704858
Depression	Phase 3	Esketamine	An open‐label, long‐term, safety and efficacy study of intranasal esketamine in treatment‐resistant depression (2015)	Completed	NCT02497287
	Phase 2 and 3	Ketamine hydrochloride	Biomarkers of response to ketamine in depression: MRI and blood assays before and after open label intranasal ketamine (2019)	Completed	NCT04216888
	Phase 4	Ketamine in combination with rTMS	Study to assess the effects of intranasal ketamine along with rTMS for patients with treatment‐resistant major depressive disorder (2020)	Unknown status	NCT04352621
	Phase 1	HS‐10345	A phase Ib, randomized, double‐blind, placebo‐controlled study evaluating the safety, tolerability and pharmacokinetics of HS‐10345 in Chinese adult subjects with treatment resistant depression (2021)	Completed	NCT05196971
	Phase 2	BPL‐003	A quadruple masked, dose‐finding study to evaluate the efficacy and safety of intranasal BPL‐003 in patients with treatment resistant depression (2023)	Recruiting	NCT05870540
	Phase 4	Brexpiprazole in combination with intranasal ketamine	A double‐blind, placebo‐controlled study of brexpiprazole plus ketamine in treatment‐resistant depression (2017)	Completed	NCT03149991
	Phase 2	Oxytocin	Testing the efficacy of intranasal oxytocin for the prevention of postpartum depression and PTSD (2015)	Completed	NCT02505984
	Phase 3	PH80 Spray	A phase 3 study of the use of PH80 for acute management of the symptoms of premenstrual dysphoric disorder (2016)	Unknown status	NCT01217775
	Phase 3	Insulin	A randomized, double‐blind, placebo‐controlled cross‐over trial evaluating the effect of intranasal insulin on depressive symptoms in individuals with major depressive disorder insufficiently responsive to antidepressant therapy (2013)	Completed	NCT00570050
Physiological synchronization	Phase 2	Oxytocin	The effects of oxytocin administration to patients and therapists on physiological synchronization: a randomized controlled pilot study (2023)	Recruiting	NCT05432089
Mood disorder	Phase 2	NPY	Intranasal administration of neuropeptide Y in healthy male volunteers (2010)	Completed	NCT00748956

Abbreviations: AD, Alzheimer's disease; AD‐SVF, adipose‐derived stromal vascular fraction; ALS, amyotrophic lateral sclerosis; BMSC, bone marrow‐derived stem cells; HD, Huntington's disease; hFGF‐1, human fibroblast growth factor 1; hUC‐MSC‐sEV‐001, human umbilical cord blood mesenchymal stem cells; MS, multiple sclerosis; MSCs, mesenchymal stem cells; NGF, nerve growth factor; NPY, neuropeptide Y; PD, Parkinson's disease; TBI, traumatic brain injury.

### 3.1 Neurodegenerative Diseases

Neurodegenerative diseases are complex disorders characterized by the progressive degeneration of neurons, triggered by various factors. Rising life expectancy has heightened the prevalence of these diseases, creating substantial medical, social, and economic challenges worldwide. Recent studies indicate that IN delivery could offer significant therapeutic advantages in both animal models and clinical trials, potentially becoming crucial in managing neurodegenerative diseases like AD, PD, amyotrophic lateral sclerosis (ALS), Huntington's disease (HD), and MS (Table 2).

**TABLE 2:** Summary of the application and function of intranasal drug delivery in neurodegenerative diseases.

Diseases	Drug	Animal model in vivo/in vitro	Intranasal condition	Mechanism of action	Nose‐to‐brain drug delivery system	References
AD	Rivastigmine	AlCl_3_‐induced rat model	12 mg/kg	Blocking the inflammatory cascade; reduction of stress process	Chitosan NPs; nanostructured lipid carrier; liposomes; mucoadhesive microspheres	[93, 94, 95, 96, 97]
	Galantamine	Scopolamine/LPS‐induced model	30 µg/µL, 20 µL	Reduction of AChE protein levels	Chitosan NPs	[98, 99, 100]
	Donepezil	/	50 µg/mL	/	Nanoemulsion; nanostructured lipid carriers	[101, 102]
	Memantine	/	5 mg/kg	/	Nanoemulsion	[103]
	Donepezil and astaxanthin	AlCl_3_‐induced rat model	0.1 mg/kg of donepezil and 0.4 mg/kg of astaxanthin	Antiamyloidogenic; antioxidation; antiacetylcholinesterase; anti‐inflammation; antiapoptosis	Nanostructured lipid carriers	[104]
	Clioquinol and donepezil	APP/PS1 transgenic mice	Donepezil (0.11 mg/kg), clioquinol (0.14 mg/kg)	Inhibition of Aβ aggregation; relief of acetylcholine‐related inflammation; protecting neurons	Human serum albumin NPs	[105]
	Rivastigmine and insulin	/	/	/	Mucoadhesive NPs	[106]
	BDNF	AD11 and WT mice	0.3, 1 and 10 µM; 0.3 mg/kg	Reduction of CD11b^+^ microglia; enhancement of neurorestorative effects	Clathrin‐NPs	[107, 108]
	Oxytocin	AlCl_3_‐induced rat model	1.25 IU/kg	Suppression of acetylcholinesterase activity, 1–42 β‐amyloid and Tau proteins levels; reduction of ERK1/2 and GSK3β	/	[109]
	Insulin	Streptozotocin‐induced rat model	2 U/day	Restoration of cerebral glucose metabolism and attenuation of astroglia activation and neuronal loss	Nanoparticulate carrier	[110]
	Aβ peptide	PDAPP mice	/	Suppression of the cerebral Aβ plaque burden and Aβ42 levels	/	[111]
	siRNA	3×Tg‐AD mice	1 mg/kg, 20 µL	Downregulation of BACE1 and caspase‐3 levels	NPs	[112]
	BACE1 siRNA	/	/	Increase in siRNA permeation through epithelial cells	Solid lipid NPs	[113]
	miR‐206‐3p antagomir	Aβ_1–42_ oligomer mice model	10 pmol	Upregulation of BDNF and activation of the BDNF/TrkB signaling pathway	MSC‐EVs	[114]
	DHA	hAPP_SwInd_ transgenic (J20) mice	10 mg/kg	/	Nanoemulsions	[115]
	NGF	AD11 anti‐NGF transgenic mice	10 µM	Rescue of recognition memory deficits	/	[116]
	TAT–haFGF	AβPP/PS1 transgenic mice	35, 100, 300 µg/kg	Improvement of cognition and reduction of Aβ plaques	/	[117]
	BACE1 siRNA and rapamycin	APPswe/PSEN1DE9 transgenic mice	437.5 µg/kg‐siRNA	Reduction of BACE1 expression and Aβ deposition, promotion of autophagy	Dual targets‐modified NPs	[118]
	Asiatic acid	Aβ_1–42_‐induced mice model	2.04 (±0.16) mg/kg/day, 30 µL	Inhibition of lipid peroxidation; suppression of TNF‐α, IL‐1β, TLR2, and TLR4 expressions	Solid lipid NPs	[119]
	Piperine	Colchicine‐induced rat model	0.045 mg/kg/day, 100 µL	Improvement of total antioxidant capacity; inhibition of AchE activity	Chitosan NPs	[120]
	18β‐Glycyrrhetinic acid	Scopolamine‐induced rat model	1 mg/kg	Elevation of CAT and SOD activities in the hippocampus	Lipid nanocapsules	[121]
	Osthole	APP/PS1 transgenic mice; scopolamine‐induced mice model	2.5 mg/kg, 50 µL; 1 µg/µL	Antioxidation; inhibition of neuron apoptosis	Borneol thermosensitive gel; microemulsion	[122, 123]
	Resveratrol	LPS‐induced AD model	20 mg/kg/day	Improvement of cognitive and memory functions, reduction of proinflammatory marker levels, and downregulation of the expression of NF‐κB and P38	Superparamagnetic iron oxide loaded chitosan coated bilosomes	[124]
	Huperzine	/	0.15 mg/kg/day	/	Lactoferrin‐modified nanoemulsion; microneedles combined with nanocarriers; surface‐modified PLGA NPs; in situ gel based on microemulsion	[125, 126, 127, 128]
	Curcumin and Berberine	Streptozocin‐induced mouse model	0.25 mg/kg	Regulation of BACE‐1 expression	Transferosomes	[129]
	Timosaponin BII	LPS‐induced AD model	20 mg/kg	Decrease in iNOS levels	Temperature/ion‐sensitive in situ hydrogel	[130]
	Luteolin	Streptozocin‐induced mouse model	2 mg/kg/day, 100 µL	Regulation of amyloid plaque formation; improvement of antioxidant effects	Chitosan NPs	[131]
	Hydroxy‐α‐sanshool	d‐Galactose‐induced AD model	1.25, 2.5, and 5.0 mg/kg	Protection of mouse hippocampal neuronal cells	Liposomes	[132]
	Ferulic acid	STZ induced in rats	80 mg/kg	Augmentation of cognitive performance	Chitosan‐coated solid lipid NPs	[133]
PD	Levodopa	Acute PD model in mice with MPTP	16 mg/kg	Improvement in behavioral and cognitive disorders; reduction of oxidative damage	NPs; lyotropic liquid crystallin	[134, 135]
	Ropinirole	/	0.05 mg/kg; 5 mg/mL	/	Mucoadhesive NPs; mucoadhesive temperature‐mediated in situ gel;	[136, 137]
	Pramipexole	Rotenone‐induced rat model	0.3 or 0.6 mg/kg	Restoration of brain dopamine levels	Thermosensitive nasal gel; chitosan NPs	[138, 139]
	Bromocriptine	Haloperidol‐induced model	3 mg/kg, 100 µL	Reduction of lipid oxidation	Niosomal; chitosan NPs	[140, 141]
	Selegiline	/	1 mg/kg	/	PLGA/lipid NPs	[142]
	Rotigotine	Human SH‐SY5Y neuroblastoma cells; haloperidol‐induced PD rats	50 µL	Decrease in lactate dehydrogenase and increase in CAT activities	Chitosan NPs: solid lipid NPs	[143, 144]
	Rasagiline	/	/	/	Chitosan‐coated PLGA NPs	[145]
	Dopamine	6‐OHDA‐induced model; SH‐SY5Y cells and 16HBE cells	/	/	Lactoferrin commodified NPs	[146]
	bFGF	6‐OHDA‐induced model	150, 300, and 600 µg/kg	Downregulation of tau phosphorylation by increasing GSK3β phosphorylation via the PI3K/Akt signaling pathway	Liposomes	[147]
	Mitochondria	6‐OHDA‐induced model	200 µg	/	/	[148]
	hUC‐MSC‐Exos	MPTP‐induced PD model	1 × 10^11^/mL, 20 µL	Neuroprotection and glia‐activated remission	/	[149]
	HEDSCs	6‐OHDA‐induced model	10^4^, 5 × 10^4^, and 10^5^ cells/µL	Increase in dopaminergic neuron marker		[150]
	Geraniol	/	1or 4 mg/kg, 10 or 40 µL 1 mg/kg, 50 µL	/	Solid lipid NPs	[151, 152]
	Naringenin	6‐OHDA‐induced model	0.72 mg/kg/d	Increased levels of GSH and SOD; reduced levels of MDA	Nanoemulsion; NPs	[153, 154]
	Rhynchophylline	SH‐SY5Y cells	20 mg/kg	Increased TH‐positive cells; decreased MDA and increased SOD, GPx levels	Thermosensitive gel	[155]
MS	Teriflunomide	Sheep nasal mucosa; cuprizone‐induced microglia activation rat demyelination model	50 µg/mL	Reduction of neuroinflammatory and risk of hepatotoxicity	Nanostructured lipid carriers	[156]
	Methylprednisolone	EAE	200 mg/kg	Reduction of infiltration into the spinal cord; downregulation Iba1 and GFAP expression; downregulation IL‐1β, IL‐6, IL‐17, IFN‐γ, and TNF‐α	/	[157]
	Dimethyl fumarate	/	/	/	Thermosensitive chitosan hydrogel	[158]
	Fingolimod	Lysolecithin‐induced demyelination model	0.3 mg/kg for 14 days 20 µL	Reduction of astrocyte activation and demyelination levels	/	[159, 160]
	Clobetasol	Cuprizone‐induced demyelination model	0.1–0.2 or 0.3 mg/kg for 10 days; 24 or 36 µL	Antioxidant and anti‐inflammatory role	Lactoferrin, chitosan double‐coated oleosomes	[161]
	MSCs	Intermittent cuprizone model	0.5 × 10^6^	Reduction of iNOS and CD86 and resumption MRC‐1 and TREM‐2; downregulation of IL‐1β and TNFα; enhancement of TGF‐β and IL‐10	/	[162]
	BMSCs	Cuprizone‐induced mouse model	A volume of 12 µL cell suspension (1 × 10^6^ cells)	Reduction of glial fibrillary acidic protein and Iba‐1; increasing Olig‐2 and APC	/	[163]
	CM‐MSC‐OL	EAE	10 µL/12 h/mouse for 2 weeks	Downregulation NLRP3, IL‐18, IL‐1β, GFAP, and Iba1	/	[164]
	MSC‐SEV	EAE	10 µg	Increase in the frequency of Foxp3+ CD25+ regulatory T cells and the level of TGF‐β	/	[165]
	A novel amnion cell secretome(ST266)	EAE	6 µL	Attenuation of visual dysfunction, and prevention of retinal ganglion cell loss; reduction of inflammation, demyelination, and retinal ganglion cell death	/	[166]
	IFN‐β	EAE	50,000 IU	Suppression neuroinflammation and demyelination; downregulation of astrocyte and microglia activation	NPs	[167]
	RA and Cal	Cuprizone mouse model; BV‐2 cell; mixed glial cell	20 µL	Downregulation of microglia cell activation	Lipid nanocapsules	[168]
	BDNF	Demyelination mouse model	/	/	Engineered exosomes	[169]
	Resveratrol	EAE	8.44 mg/kg; 3 mg/kg, 30 µL	Reduction of RGC loss and inflammatory responses	NPs; macrophage exosomes	[170, 171]
	Astragaloside IV	16 HBE and BV‐2 cells; EAE	1.3 mg/kg/day	Downregulation of astrocyte and microglial activation; reduction of demyelination; increasing of remyelination	β‐Asarone‐modified chitosan NPs	[172]
HD	siRNA	Female YAC128 transgenic mice	24 µL	/	Chitosan NPs	[173]
	Rosmarinic acid	/	12 mg; 14 days	/	Solid lipid NPs	[174]
ALS	N‐acetyl‐l‐cysteine	G93A mutant transgenic mice	20 µL	/	Cell‐penetrating peptide‐modified polymer nanomicelles	[175]
	MSC‐derived sEVs	G93A–SOD1 transgenic mice	3.0 ×1 0^9^	Reduction of spinal motoneuron death and synaptic denervation; alleviation of mitochondrial damage	/	[176]
	Insulin and NOS inhibitor	SOD1^G93A^ mice	0.87 U	/	/	[177]
	NGF	G93A–SOD1 transgenic mice	50 µg/mL	Increase in the quantity of TrkA receptors	/	[178]
	HT‐HPL	SOD1^G86R^ mouse	20 µL	/	/	[179]
	NPY	SOD1^G86R^ mouse	150 µg/kg, 20 µL	/	/	[180]

Abbreviations: AChE, acetylcholinesterase; AD, Alzheimer's disease; AF‐NSC‐EV, amniotic fluid‐derived neural stem cell‐derived extracellular vesicle; APC, adenomatous polyposis coli; ATN, athymic nude; Aβ, β‐amyloid; BACE1, beta‐secretase 1; BBB, blood–brain barrier; BDNF, brain‐derived neurotrophic factor; BMSCs, bone marrow‐derived mesenchymal stem cells; Cal, calcitriol; CAT, catalase; CCI, controlled cortical impact; CM‐MSC‐OL, conditioned medium derived from MSCs‐differentiated oligodendrocytes; DHA, docosahexaenoic acid; EAE, experimental autoimmune encephalomyelitis; E‐NSC‐EV, neonatal enteric neural stem cell‐derived extracellular vesicle; FoxO, forkhead box O; GFAP, glial fibrillary acidic protein; GPx, glutathione peroxidase; GSH, glutathione; H9‐NSC‐EV, H9 cell‐induced neural stem cell‐derived extracellular vesicle; HAND, HIV‐associated neurocognitive disorders; HD, Huntington's disease; HEDSCs, human endometrium‐derived stem cells; HNC, hippocampal neuronal cells; hNSC‐EV, human neural stem cell‐derived extracellular vesicle; HT‐HPL, heat‐treated human platelet lysate; hUC‐MSC‐Exos, human umbilical cord mesenchymal stem cell exosomes; Iba1, ionized calcium‐binding adapter molecule 1; IFN‐β, interferon‐beta; IFN‐γ, interferon‐gamma; IGF‐NSC‐EV, insulin‐like growth factor‐1‐neural stem cell‐derived extracellular vesicle; IL, interleukin; IL‐18, interleukin‐18; IL‐1β, interleukin‐1 beta; IS, ischemic stroke; MDA, malondialdehyde; MPTP, 1‐methyl‐4‐phenyl‐1,2,3,6‐tetrahydropyridine; MRC‐1, mannose receptor c‐type 1; MS, multiple sclerosis; MSCs, mesenchymal stem cells; NF‐κB, nuclear factor‐kappa B; NGF, nerve growth factor; NLRP3, NLRP family pyrin domain containing 3; NPY, neuropeptide Y; Olig‐2, oligodendrocyte lineage transcription factor‐2; PA, insulin and palmitic acid; PD, Parkinson's disease; RA, retinoic acid; SDF‐1α, stromal cell‐derived factor‐1 alpha; SOD, superoxide dismutase; STZ, streptozocin; TGF‐β, transforming growth factor‐beta; TLR2, toll‐like receptor 2; TLR4, toll‐like receptor 4; TNF‐α, tumor necrosis factor‐alpha; TRE‐2, triggering receptor expressed on myeloid cells 2; TrkA, tropomyosin receptor kinase A.

#### 3.1.1 Alzheimer's Disease

AD is a neurodegenerative condition that progressively worsens. It involves memory loss, cognitive decline and behavioral changes. The pathogenesis of the disease involves multiple factors, such as genetic changes, β‐amyloid (Aβ) protein deposition, tau accumulation into neurofibrillary tangles in neurons, and abnormal tau phosphorylation [181, 182]. In Alzheimer's therapy, NBDD act as an important drug delivery strategy, facilitating the transfer of the drug to the brain and brain targeting. Excitingly, chemical drugs, biologics, and herbal components can be used to by NBDD.

The chemical drugs including cholinesterase inhibitors (donepezil, rivastigmine, galantamine) and N‐methyl‐d‐aspartate receptor antagonists (memantine) have been reported to be administered by IN. Based on innovative nanomaterials for IN delivery, rivastigmine‐loaded chitosan NPs and nanostructured lipid nanocarriers, demonstrate controlled release, enhanced penetration, and decreased acetylcholinesterase (AChE) expression. These properties underscore their potential in managing AD by decelerating disease progression, inhibiting inflammation, and mitigating oxidative stress [93, 94]. A separate investigation revealed that NBDD of rivastigmine liposomes improved drug distribution and retention within the CNS, particularly in the hippocampus and cortex [95]. Similarly, IN galantamine loaded in NPs could enhance brain delivery, delay elimination, and improve brain targeting, significantly reducing AChE protein levels and activity in rat brains, thus surpassing conventional galantamine therapy in an AD rat model [98, 183]. Donepezil [101] and memantine [103] formulations have also been proposed as novel IN approaches for Alzheimer's treatment. Recent studies have reported the advantageous effects of dual‐drug IN delivery in addressing multiple facets of AD pathology. Donepezil and astaxanthin were coformulated using nanostructured lipid carriers via the NBDD, demonstrating significantly enhanced antiamyloid, antioxidant, anti‐AChE, anti‐inflammatory, and antiapoptotic activities [104]. Simultaneously, clioquinol and donepezil were coencapsulated in human serum albumin NPs, achieving high brain uptake and prolonged retention via NBDD, which effectively inhibited Aβ aggregation, mitigated acetylcholine‐related inflammation in microglial cells, and protected primary neurons from Aβ‐induced neurotoxicity in vitro [105]. Additionally, a new study highlights the potential of rivastigmine and insulin‐loaded mucoadhesive NPs for IN delivery as a promising strategy [106].

Biologics is one of the major therapeutic for AD. Biologics, including proteins, peptides, and antibodies, face challenges with traditional delivery methods, but the IN delivery offers significant advantages by overcoming these limitations, particularly for large molecules [184]. Among these brain‐derived neurotrophic factor (BNDF), which is essential for neuronal survival and plasticity. IN of BDNF has shown to alleviate memory deficits and reduce microglial proliferation in AD mice, though it does not impact Aβ accumulation or tau hyperphosphorylation, suggesting its action primarily improves cognitive function by modulating synaptic plasticity [107]. The neuropeptide oxytocin also shows therapeutic potential in AD. Research has indicated that IN oxytocin reduces AChE and Aβ levels, along with decreases tau and other neuroinflammatory markers, further improving cognitive function and offering neuroprotection [109]. However, clinical trials have not yet yielded sufficient evidence to confirm significant therapeutic effects, although its favorable safety profile warrants further investigation [185]. Another promising molecule is insulin, which has shown neuroprotective effects through IN delivery, whose mechanisms are related to influence Aβ clearance, tau phosphorylation, and synaptic plasticity [186, 187, 188, 189]. Despite initial clinical trial results showing no significant cognitive benefits [190], a phase II trial showed that IN insulin may alter inflammation, improve cerebrospinal fluid biomarkers, and slow symptom progression [191]. Moreover, Aβ peptide has been used in IN delivery studies aimed at reducing Aβ fibrils and mitigating microglial and astrocytic activation, but the clinical trials were terminated due to cases of meningoencephalitis observed in some patients [111, 192]. Recently, the potential of gene therapy, specifically small interfering RNA (siRNA) technology, has also been explored [193]. siRNA NPs, when administered intranasally, have demonstrated the ability to cross the nasal mucosa and reach affected brain areas, which could downregulate key enzymes like BACE1 and caspase‐3, and reduce neurological injury in AD mouse models [112, 113].

In addition, researches have shown that IN administration of various compounds, such as Asiatic acid in solid lipid NPs, piperine‐loaded chitosan NPs, and 18β‐glycyrrhetinic acid in lipid nanocapsules, could mitigate tau hyperphosphorylation, prevent astrocyte and microglial activation, decrease IL‐1β, TNF‐α, and MDA levels in the brain, and improve cognitive function in AD models [119, 120, 121]. Similarly, studies have confirmed that NBDD of osthole/borneol thermosensitive gels, and superparamagnetic iron oxide‐loaded resveratrol enhanced brain bioavailability, inhibited neuronal apoptosis, lowered Aβ levels by inhibiting BACE1, reduced proinflammatory markers and improved cognitive functions [122, 124]. Importantly, it has been found that NBDD of lactoferrin‐modified nanoemulsion, nanocarriers, NPs, and in situ gel to enhance the targeting of Huperzine A to the brain [125, 126, 127, 128]. Additionally, the compounds such as curcumin, berberine, and timosaponin BII are emerging as promising therapeutic candidates via NBDD for AD [129, 130, 194].

#### 3.1.2 Parkinson's Disease

Parkinson's disease is a neurodegenerative condition caused by the degeneration of dopaminergic neurons in the substantia nigra, resulting in lower dopamine (DA) levels and compromised muscle control. Its pathogenesis involves factors such as α‐synuclein aggregation, oxidative stress, mitochondrial dysfunction, and defective autophagy [195, 196].

Levodopa, the preferred PD treatment, has its limitations due to poor brain uptake. However, using levodopa‐loaded NPs via NBDD could effectively improves its brain concentration and accelerates its action [134]. A nasal spray utilizing lyotropic liquid crystalline in situ gel has been developed to extend levodopa retention within the nasal cavity and mitigate oxidative stress in PD [135]. The potential of nasal formulations of DA receptor agonists, such as pramipexole, ropinirole, and bromocriptine, is currently under investigation. The IN delivery of ropinirole in situ gel formulations or mucoadhesive NPs has been shown to enhance drug delivery to the brain and improve therapeutic outcomes [136, 137]. Similarly, the pharmacodynamic results highlighted that pramipexole dihydrochloride‐loaded chitosan NPs and pramipexole gel markedly enhanced antioxidant status by increasing SOD and CAT activities, while also raise brain DA levels and restore locomotor function [138, 139]. Bromocriptine mesylate niosomes have been found to be safe for IN administration, displaying a significant enhancement in brain distribution and improved pharmacodynamic behavior with a 10‐fold dose reduction [140]. In addition, chitosan NPs play a crucial role in the delivery of bromocriptine, enhancing brain‐targeting efficiency [141]. Apart from these, therapeutic effect has been confirmed by direct nose‐to‐brain transport of the IN formulation of selegiline [142].

Current synthetic drugs are limited in halting the progression of PD. In contrast, biological agents, which involves gene therapy and stem cell therapy, are emerging as promising treatments for PD. DA‐loaded NPs modified with borneol and lactoferrin improved drug delivery for PD, reduced side effects, and alleviated brain damage in rats, enhanced behavioral and neurotransmitter outcomes [146]. In another study, plasmid DNA NPs encoding human glial cell line‐derived neurotrophic factor significantly enhanced gene expression, neuron protection, and reduced the severity of brain lesions in a 6‐hydroxydopamine (OHDA)‐induced PD model [197]. Similarly, IN administration of bFGF encapsulated in liposomes reduced tau phosphorylation, alleviated behavioral dysfunction in the same model [147]. Moreover, urocortin, a corticotropin‐releasing factor‐related peptide, incorporated into odorranalectin‐conjugated NPs, significantly enhanced brain delivery and neuroprotective effects [198]. NBDD of a self‐targeting nanocarrier, PR‐EXO/PP@Cur, integrates mesenchymal stem cells (MSCs)‐derived EVs with curcumin to provide a three‐pronged synergistic approach for treating the complex pathologies of PD by reduced α‐synuclein aggregates, enhanced neuronal function, and reduced neuroinflammation [199].

Herbal components such as geraniol, curcumin, naringenin, and rhynchophylline are important members of drugs for the treatment of PD, which have been extensively studied in the development of IN delivery. For instance, the NBDD of geraniol, in combination with a ursodeoxycholic acid lipid formulation, has been shown to preserve the structural integrity of the nasal mucosa, suggesting potential advantages in drug delivery systems for PD [151]. Furthermore, β‐cyclodextrin and its derivative could also enhance geraniol bioavailability [152]. Recent studies have demonstrated IN administration of vitamin E‐loaded naringenin nanoemulsion [153] or NPs [154] could significantly elevate the levels of GSH and SOD. This results in enhanced neuroprotective and antioxidant effects against 6‐OHDA‐induced neurotoxicity in SH‐SY5Y cells. Additionally, a curcumin analogue‐based nanoscavenger has been identified as an IN delivery system capable of controlled‐release properties [200]. Rhynchophylline thermosensitive gel, when applied by IN delivery, reduced MDA levels, boosted SOD and GPx activities, and promoted the regeneration of TH‐positive cells in the substantia nigra and striatum, contributing to repair motor dysfunction and protect dopaminergic neurons in PD [155].

#### 3.1.3 Multiple Sclerosis

MS a demyelinating, inflammatory, and neurodegenerative disorder affecting the CNS. Optic neuritis, a common symptom of MS, frequently leads to permanent vision impairment due to retinal ganglion cell damage [201]. Several US FDA‐approved drugs for MS treatment include teriflunomide [156], methylprednisolone [157], fingolimod [159], dimethyl fumarate [158], and clobetasol [161]. As current studies indicate, these drugs primarily work by reduced central inflammation and demyelination levels in MS patients through IN delivery. It appears that the IN administration of methylprednisolone was as efficient as the intravenous route in treating neuroinflammation in experimental autoimmune encephalomyelitis (EAE), and no damage to the nasal cavity was found [157]. It is intriguing that a thermosensitive chitosan hydrogel was loaded with dimethyl fumarate, which confirmed its role in reduced side effects, increased adherence to therapy, and improved bioavailability in the brain [158]. Furthermore, the lactoferrin–chitosan double‐coated oleosomes, which were loaded with clobetasol, highlight the significant antioxidant and anti‐inflammatory capacity of the formulation and succeed in remyelination with a 6.6‐fold reduction in drug dose compared with previous studies [161]. Cell therapy, such as adipose‐derived MSCs [202], MSCs [162] and MSCs‐EVs [165] has been under investigation for MS treatment by NBDD. When MSCs were administered intranasally, they reshaped macrophage polarity along with modifying glial and inflammatory responses, thereby favoring therapy [162]. A study showed that IN delivery of SDF‐1α‐preconditioned BMSCs enhances remyelination in a cuprizone‐induced mouse model of MS [163]. NBDD of conditioned medium derived from MSCs‐differentiated oligodendrocytes has been suggested to reduce inflammation, enhance remyelination, and improve neurological behavior in EAE mice [164]. In addition, a series of IN biologic, such as a novel amnion cell secretome (ST266) [166], anticaspase‐1 therapy [203], IFN‐β [167], BDNF [169], retinoic acid, and calcitriol [168], have been suggested as promising strategies for MS therapies. A brain‐targeted engineered exosome‐mediated BDNF has recently become a highly effective delivery route to the brain, which has a significant therapeutic effect on remyelination [169]. The combination of retinoic acid and calcitriol, which were encapsulated in lipid nanocapsules, stimulates oligodendrocyte progenitor cell differentiation in vitro and in vivo after IN administration [168].

Given that herbal therapies are a natural, safe, and reliable remedy for the treatment of neurodegenerative diseases, they represent a promising therapeutic approach for MS [204]. Studies indicated that resveratrol‐loaded macrophage exosomes and NPs, delivered by IN, are essential for targeting CNS microglia, where they inhibit inflammation and enhance neuroprotection [170, 171]. Astragaloside IV, a triterpenoid saponin, is used to treat MS and has been suggested to be delivered by β‐Asarone‐modified chitosan NPs due to its ability to suppress inflammatory infiltration, reduce demyelination, and promote remyelination in a mice EAE model [172]. In addition, there is a wealth of herbal resources that can be explored for the treatment of MS through IN administration, representing a promising direction for future research.

#### 3.1.4 Huntington's Disease

HD is a neurodegenerative disorder characterized by motor dysfunction, behavioral and psychiatric symptoms, and cognitive decline [205]. Recent advancements have demonstrated that IN administration of Neuropeptide Y (NPY) can reduce mutant Huntingtin aggregation by enhancing BDNF levels and mitigating microglial activation [206]. Additionally, IN delivery of anti‐HTT siRNA, modified with chitosan‐based NPs to safeguard the payload from degradation, has shown efficacy in reducing HD gene expression [173]. Nevertheless, the current body of research on IN therapeutics for HD remains limited, necessitating further development of potential treatment strategies for effective management of the disease.

#### 3.1.5 Amyotrophic Lateral Sclerosis

ALS is a terminal neurodegenerative disorder characterized by progressive muscle weakness, atrophy, and paralysis, primarily targeting the motor neurons within the brain and spinal cord that are essential for muscle movement. NBDD therapies employ various drugs to combat ALS, including N‐acetyl‐l‐cysteine [175], MSC‐derived sEVs [176], nerve growth factor (NGF) [178], heat‐treated human platelet lysate preparations [179] and NPY [180]. Studies have shown that IN NGF administration, along with lateral ventricle neural stem cell grafts, synergistically enhances TrkA receptor expression, promotes the migration of exogenous neural stem cells, stimulates proliferation in neurogenic regions of the brain, and aids in the preservation of motor neurons within the spinal cord [178]. After IN administration of MSC‐derived sEVs, significant therapeutic advancements have been observed in addressing motor neuron loss, mitochondrial dysfunction, axonal demyelination, synaptic denervation, and neuromuscular junction degeneration [176].

Taken together, previous studies collectively indicate that the NBDD strategy holds promise as a therapeutic approach for neurodegenerative diseases, particularly in enhancing drug delivery to the brain and minimizing systemic side effects. Nonetheless, significant challenges persist, notably in terms of technical difficulties and drug absorption. Neurodegenerative diseases such as AD and PD involve intricate neural mechanisms, rendering IN administration alone inadequate for addressing all symptoms. Consequently, future research should prioritize the development of multidrug or multimodal IN therapies. Thus, intensified efforts are essential to address these challenges and facilitate the translation of NBDD strategies into clinical practice.

### 3.2 Acute Neurological Diseases

The treatment of acute neurological disease focuses primarily on reducing neuroinflammation and promoting neuroprotection and regeneration. In contrast to therapies for neurodegenerative diseases, IN delivery for acute neurological diseases has garnered increasing attention and researched as a potential therapeutic strategy. It can be used in the treatment of several acute or sudden‐onset neurological conditions, such as: stroke and TBI (Table 3).

**TABLE 3:** Summary of the application and function of intranasal drug delivery in acute neurological diseases.

Diseases	Drug	Animal model in vivo/in vitro	Intranasal condition	Mechanism of action	Nose‐to‐brain drug delivery system	References
Stroke	Progesterone	MCAO model	8 mg/kg	Decrease in neurological deficits, infarct, neuronal loss, and early BBB disruption	Lipophilic gel	[207, 208]
	Edaravone and borneol	tMCAO/R model	3 mg/kg, 6 mg/kg; 20 µL	Alleviation of neurological deficit symptoms	Temperature‐sensitive hydrogels	[209]
	Nestorone	MCAO model	0.08 mg/kg; 1 µL/nostril/10 g	Decrease in infarct size and improvement in functional outcomes	Oleogel	[210]
	Deferoxamine	MCAO model	6 mg	Increase in brain exposure and protection in rat ischemic stroke	/	[211]
	Dexamethasone	MCAO model	0.25 mg/kg	Reduction in mortality, neurological deficits, infarct volume size, BBB permeability, inflammatory cell infiltration, and glial activation	/	[212]
	CRISPR/Cas9	pMCAO model	100 µL	Upregulation of the target gene Sirt1	NPs	[213]
	HFSCs	MCAO model	2 × 10^6^, 100 µL	Decrease in infarct volume; increase in NeuN and VEGF expression; prevention of BDNF and neurotrophin‐3 overexpression	/	[214]
	hNSCs	pMCAO model	1 × 10^6^ cells in 10 µL PBS	Reduction in lesion volumes	/	[215]
	Bone marrow stromal cells	/	1 × 10^6^ cells/100 µL; 100 µL	Increase in NeuN/BrdU and Glut‐1/BrdU double‐positive cells, neurogenesis, and angiogenesis	/	[216]
	BMSCs	FIS model of mice	100 µL (1 × 10^6^ cells)	Suppression of cell death	/	[217]
	BMSCs and IGF‐1	FIS model of mice	IGF‐1 (500 ng), 1 × 10^6^ cells	Upregulation of the protein levels of BDNF, VEGF and Ang‐1; promotion of local cerebral blood flow	/	[218]
	hBMSCs‐derived EVs	MCAO	2.4 × 10^9^ EVs	Improvement of neurological function	/	[219]
	hAT‐MSC‐derived EVs	/	200 µg/kg	Improvement of the BBB, and restabilization of vascularization; reduction of infarct volume	/	[220]
	MSCs	/	5 × 10^4^ cells; 50 µL	Reduction of NO content	/	[221]
	ADSC‐Exo	MCAO model	10 µg	Inhibition of ferroptosis by targeting CHAC1 in neurons	/	[222]
	Anti‐miR‐181a oligonucleotide	MCAO model	25 µL	Upregulation of Bcl‐2 expression; reduction in TNF‐α expression and apoptosis; knockdown of MiR‐181a	Anti‐RAGE exosomes	[77]
	siRNA	MCAO model	/	Reduction in neuronal cell death by effective inhibition of Fas signaling and prevention of cytochrome *c* release	Fas‐blocking peptide 9R carrier system	[223]
	NMNAT1	MCAO model	3 µg/kg, 10 µg/kg, 30 µg/kg, 10 µL	Prevention of tight junction protein loss; decrease in acetylated NF‐κB, p53, MMP‐9 levels, and cell apoptosis; protection of BBB integrity via the NAD+/SIRT1 signaling pathway	/	[224]
	Nucleotides	HepG2 cells; MCAO model	/	Enhancement of cell survival and reduction of infarction in energy‐/oxygen‐depleted environments	pH‐sensitive bioenergetic nucleotide NPs	[225]
	microRNA	MCAO model	5 µg/mL	Improvement of neuroprotective effects	RVG29‐modified NPs	[226]
	Melatonin	CIRI model; sheep nasal mucosa	50 µL	Lowering of oxidative stress; decrease in TNF‐α, NO and MPO levels and caspase‐3, cytochrome c, Bax activity; upregulation of Bcl‐2	Lipidic nanocapsules; core–shell polymeric nanocapsules	[227, 228]
	Guanosine	FPI	30 mg/mL	Neuroprotective effect	/	[229, 230]
	NR2B9c peptide	Calu‐3 cells; MCAO model	0.3 mg/kg	Protection of neurons against excitotoxicity; ameliorating neurological function deficits	Wheat germ agglutinin‐modified NPs	[231]
	Fas‐blocking peptide	MCAO model	2 mg/kg	Reduction of neuronal cell death	/	[232]
	Apelin‐13	/	4 mg/kg	Upregulation of APLNR, and Bcl‐2; decrease in TNF‐α, IL‐1β, MIP‐1α, MCP‐1 levels, and caspase‐3 activation; reduction in microglia recruitment and activation; increase in VEGF and MMP9 expressions	/	[233]
	Caspase‐1 Inhibitor	4‐vessel occlusion model	20 µL	Inhibition of caspase‐1 activity, reactive gliosis, and neuroinflammatory; attenuation of the caspase‐3‐dependent apoptotic pathway	/	[234]
	Insulin	Autologous blood‐induced hemorrhage model	0.5, 1, or 2 IU, 10 µL	Increase in p‐AKT and p‐GSK3β levels; decrease in BBB permeability	/	[235]
	G‐CSF	MCAO model	60 µg/kg, 50 µL	Promotion of angiogenesis and neurogenesis	/	[236]
	IGF‐1	MCAO model	75 µg	Reduction in infarct volume, cerebral edema, and neurologic deficits	/	[237]
	NGF	MCAO model	100 µL	Enhancement of neurogenesis	/	[238]
	BDNF	tMCAO model	1.0 × 10^10^	Increase in neurogenesis, angiogenesis, synaptic plasticity, and fiber preservation; increase in neuroprotection and reduction in inflammation; activation of the BDNF/TrkB signaling	Small extracellular vesicles	[239]
	Human IL‐1RA	MCAO model	50 mg/kg, 100 µL	Downregulation of TNF‐α and IL‐1β expression	/	[240]
	IL‐1b antagonist	CCI‐induced TBI rat model	1 mg/kg	Increase in GSH level, activity of GR, GP, GT, and HSP70 expression	RAIL gel	[241]
	Oxytocin	tMCAO model	8 IU/mouse	Reduction in the number of dead nerve cells and TNFα, IL‐1β levels	/	[242]
	Exendin‐4	MCAO model	20 µL	Decrease in caspase‐3; modulation of the cAMP/PKA and PI3K/Akt pathways	/	[243]
	bFGF	MCAO model	30 µL, 30 µg	Enhancement of progenitor cell proliferation	/	[244]
	aFGF	MCAO model	200 µL (20 µL/drop)	Improvement of neurological functional recovery	/	[245]
	rhEPO	MCAO; CCI‐induced TBI rat model	0.6, 2.4, 6, 12 U/20 µL; 2. 50 U/100 g/week; 500, 1000, 2500 IU/kg, 60 µL	Reduction of infarct volume, brain swelling, and cell damage	/	[246, 247, 248]
	Dynorphin A (1–8)	MCAO model	0.1 nmol/mL or 500 nmol/mL 0.1 mL	Reduction of ischemia‐induced apoptosis; suppression of oxidative stress	PCG–arginine NPs	[249, 250]
	PACAP	pt‐MCAO model	1 µg/µL to 1 fg/µL	/	/	[251]
	Mitochondria	pt‐MCAO model	100 µg	Activation of AMPK and SIRT1/PGC‐1α signaling pathway; inhibition of NLRP3 inflammasome activation; improvement of neurological dysfunction	/	[252]
	β‐1, 3‐galactosyltransferase 2	MCAO model	0.01, 0.02, 0.04 µg/kg, 10 µL	Attenuation of NF‐κB, IL‐6, TNF‐α, and IL‐1β; inhibition of NLRP3 inflammasome; alleviation of BBB permeability; improvement of neurological function, neuron apoptosis	/	[253]
	OPN	Post‐MCAO model	1 µg/rat; 25 ng/µL, 20 µL	Suppression of activation of microglial cells, augmentation of M2 polarization and levels of IL‐1β, IL‐6, and TNF‐α;	Gelatin NPs	[254, 255]
	Wnt‐3a	tMCAO model	2 µg/kg; 1.2 µg/kg; 2 mg/kg/day; 25 µL	Attenuation of TUNEL‐positive cells, Iba‐1 GFAP, IL15 protein levels; elevation of IL33 protein; Downregulation of iNOS, TNF‐α and GFAP/C3‐positive cells; upregulation of the expression of BDNF.	/	[256, 257, 258]
	Baicalin, borneol, and cholic acid	MCAO model	9 mg/kg	Improvement of neurological deficits, cerebral pathology; modulation of PI3K/Akt and MAPK signaling pathway	Liposomes	[259]
	Salvinorin A	tMCAO middle cerebral artery	50 µg/kg,10 µL; 2.5 µL/kg	Decrease in proinflammatory microglia/macrophages; attenuation of the infiltration of peripheral immune cells; protection of the integrity of the BBB;	/	[260, 261]
	Thymoquinone	MCAO model	10 mg/kg	/	Mucoadhesive nanoemulsion	[262]
	Rg3 and PNS	MCAO model	3.6 mg/kg Rg3, 13.2 mg/kg PNS	Targeting of ischemic sites, including microglia	Macrophage membrane‐cloaked liposome	[263]
	Volatile oil of Chaxiong	MACO model	13.29 mg/kg,50 μμ	Reduction of the neurological deficit score	Nanoemulsions in situ gel	[264]
	Catalpol	MCAO model	1 mg/kg	Increase in SOD and decrease in MDA level; promotion of the expression of Bcl‐2 protein; activation of Nrf 2/HO‐1 pathway; reduction of oxidative stress and apoptosis	/	[265]
	Cimicifugoside H‐1	MCAO model	5 mg/kg	/	/	[266]
	Resveratrol	MCAO model	50 mg/kg	Reduction of MMP‐9 and NF‐kB expression levels; attenuation of neurological deficits, brain edema, and BBB disruption	/	[267]
	Scutellarin	/	4 mg/kg, 25 μμ	/	HP‐β‐CD/chitosan NPs	[268]
	Breviscapine	/	20 mg/kg, 100 μμ	/	Nanosuspension in situ gelling system	[269]
	Rutin	MCAO model	10 mg/kg	Improvement of neurobehavioral activity and reduction of infarction volume	Chitosan NPs	[270]
	Salvianolic acid B	/	50 mg/kg	Reduction in the number of newborn nerve cells	/	[271]
	Eugenol	/	10 mg/kg	/	Chitosan‐coated‐PCL‐NPs	[272]
TBI	Lidocaine		Initial dose was 1 mg/min, gradually increased to 2 mg/min	/	/	[273]
	SUR1 inhibitor glibenclamide	/	/	Reduction of brain edema assessment and prevention of brain damage	/	[274]
	ALM solution	Primary human nasal cell	Adenosine 1 mM, lidocaine 3 mM, Mg^2+^ 2.5 mM	/	/	[275]
	tPA	CCI model	600 µg/rat	Increase in neuroblasts and neurogenesis; promotion of midline‐crossing CST axon sprouting; increase in mature BDNF protein level	/	[276]
	LMNSC008	CCI model	1 × 10^6^ cells in 24 µL	Reduction of inflammatory response, microglial function	/	[277]
	hMSC‐EVs	CCI model	6.4, 12.8, or 25.6 × 10^9^ EVs/mice	Prevention of dysregulation of the BDNF–ERK–CREB signaling pathway	/	[278]
	hASC‐Exo	CCI model	10 µg	Reduction of cortical brain injury and microglial activation; alleviation of oxidative stress	/	[279]
	hPSC‐derived GPC‐EVs	TBI model	2 × 10^11^ particles/mL; total protein concentration 12.7 ± 0.6 µg/mL	Reduction of expression levels of IL‐6, IL‐1β, IL‐12; increase in protein levels of NF‐κB	/	[280]
	PEVs	CCI; human SH‐SY5Y neuroblastoma cells; BV‐2 cells	60 µL or 1.2 × 10^8^	Exertion of anti‐inflammatory activity	/	[281]
	NGF	Young rats	5 µg/d	Reduction of tau hyperphosphorylation; decrease in GSK‐3β activation; lowering of IL‐1β and NF‐κB	/	[282, 283]
	Insulin	CCI model	6 IU/day, 60 µL; 1 IU, 10 µL	Decrease in hippocampal lesion volume and less microglial immunolabeling; promotion of neurological recovery; reduction of microglia activation and concentration of IL‐1β or TNF‐α; suppression of autophagy; activation of the PI3K/AKT/mTOR signaling pathway	/	[284, 285, 286]
	Oxytocin	eCCI model	/	/	/	[287]
	Human platelet lysate	CCI model	20 µL	Mitigation of cortical neuroinflammation and synaptic impairments; enhancement of antioxidant defense	/	[288]
	Agomir‐let‐7i	HSI and PVD Stroke	24 µL	Suppression of neuroinflammation and glial scar formation; reduction of neuronal apoptosis	/	[289]
	KAFAK peptide	Primary rat brain microvascular endothelial cells; mFPI model	6 µL	Reduction of IL‐1β and IL‐6 levels	/	[290]
	Antisecretory peptide AF‐16	mFPI model	2 mg/mL, 25 µL	/	/	[291]
	NAD	/	20 mg/kg, 60 µL	Restoration of NAD+ levels; inhibition of microglial activation	/	[292]
	Wnt3a	CCI model	2 µg/kg; 25 µL	Suppression of autophagic activity; reduction of autophagic and injury; increase in levels of β‐catenin, GDNF, and VEGF	/	[293]
	OPN	CCI model	5 µg/µL, 10 µL	/	/	[294]
	IL‐4	CCI model	/	Increased expression of PPARc and arginase‐1 within Mi/MU	NPs	[295]
	Elovanoids	FPI model	1 µg/µL; 20 µg/rat	/	/	[296]
	Paclitaxel	Mild closed‐skull TBI	0.6 mg/kg	/	/	[297]
	Icariin	FPI model	/	/	HP‐β‐cyclodextrin	[298]

Abbreviations: ADSC‐Exo, adipose‐derived mesenchymal stem cells; aFGF, acidic fibroblast growth factor; ALM, adenosine, lidocaine and Mg2+; bFGF, basic fibroblast growth factor; BMSC, bone marrow mesenchymal stem cell; BMSCs, bone marrow mesenchymal stem cells; BrdU, bromodeoxyuridine; CCI, chronic cerebral ischemia; CCI, controlled cortical impact; CHAC1, cation transport regulator‐like protein 1; CI, cerebral ischemia; CIRI, cerebral ischemia/reperfusion injury; eCCI, electroconvulsive shock injury; eCCI, electronic controlled cortical impact; FCI, focal cerebral ischemia; FIS, focal ischemia stroke; GCI, global cerebral ischemia; G‐CSF, granulocyte colony‐stimulating factor; GP, glutathione peroxidase; GPC‐EVs, glial cell extracellular vesicles; GR, glutathione reductase; GSH, glutathione; GT, glutathione transferase; hASCexo, human adipose‐derived stem cell exosomes; hAT‐MSC‐derived EVs, human adipose tissue mesenchymal stem cell‐derived extracellular vesicles; HFSCs, hair follicle‐derived stem cell; hMSC‐EVs, human mesenchymal stem cell‐derived extracellular vesicles; hNSCs, human neural stem cells; hPSC‐derived GPC‐EVs, human pluripotent stem cell‐derived GPC‐EVs; HSI, hippocampal stab injury; IL, interleukin; IS, ischemic stroke; LMNSC008, L‐myc immortalized human neural stem cells; MAPK, mitogen‐activated protein kinase; MCAO, middle cerebral artery occlusion; mFPI, mild forced head injury; MMP9, matrix metallopeptidase 9; MMP‐9, matrix metallopeptidase 9; MPO, myeloperoxidase; NAD, nicotinamide adenine dinucleotide; NeuN, neuronal nuclei; NF‐κB, nuclear factor‐kappa B; NGF, nerve growth factor; NMNAT1, nicotinamide mononucleotide adenylyltransferase 1; OPN, osteopontin; PACAP, pituitary adenylate cyclase‐activating polypeptide; PEVs, platelet extracellular vesicles; PNS, panax notoginseng saponins; post‐MCAO, postmiddle cerebral artery occlusion; pt‐MCAO, permanent middle cerebral artery occlusion; PVD, pial vessel disruption; Rg3, ginsenoside Rg3; rhEPO, recombinant human erythropoietin; SOD, superoxide dismutase; TBI, traumatic brain injury; TNF‐α, tumor necrosis factor‐alpha; tPA, tissue plasminogen activator; VEGF, vascular endothelial growth factor.

#### 3.2.1 Stroke

Although stroke is a neurological injury resulting from the obstruction of cerebral blood flow, it has received insufficient attention [299]. Since IN delivery of chemical, biologicals, and herbal components holds promise as a potential drug delivery method, it may offer a viable treatment pathway. IS are more common, but hemorrhagic strokes lead to higher rates of mortality and disability [300].

Thus, the IN delivery of chemical drugs—such as dexamethasone, edaravone, progesterone, deferoxamine, and nestorone—may offer an effective, brain‐targeting formulation, providing a viable option for the clinical prophylaxis and treatment of IS. In a pilot study, IN delivery of progesterone resulted in sustained brain drug levels for over 24 h, decreased corticosterone levels, and reduced early BBB disruption [207]. Its protective effects were dose‐dependent, with 8 mg/kg of progesterone providing strong, long‐lasting cerebroprotection [208]. Moreover, a single dose IN delivery of dexamethasone (0.25 mg/kg) was the most effective conditions to treat neuroinflammation in MCAO mice [212]. It is interesting that sex differences in the response to nestorone IN treatment were found, showing as improving functional outcomes and reducing ischemic lesions in male mice, but having no effect in female mice at 48 h post‐MCAO [210]. Apart from this, deferoxamine may act as a prophylactic for stroke‐prone patients, because the pretreatment using three times of 6 mg IN doses in 48 h before MCAO could effectively reduce infarct volume by 55% compared with controls [211].

Biological agents are also relevant to treatments for stroke through IN delivery, particularly including cell therapy, nucleic acid and protein, which exert therapeutic effects through neuroprotection, reducing inflammation, and promoting nerve repair. The protein‐based CRISPR–dCas9 system plays a crucial role in targeting neuroprotective genes in general. The IN administration of the dCas9/CaP/PEI–PEG–bHb NPs upregulates Sirt1 in the brain, reduces cerebral edema, and improves survival after permanent MCAO [213]. On one hand, up until now, different cell types and their derived EVs, such as hair follicle‐derived stem cells [214], human neural stem cells [215], bone marrow stromal cells [216], MSCs [221], bone marrow MSCs [217], human bone marrow MSCs‐derived EVs [219], adipose‐derived MSCs‐exosomes [222], and human adipose tissue MSC‐derived EVs [220], have been utilized to treat stroke through IN administration in animal models. Coapplied IN delivery of IGF‐1 protected BMSCs from apoptosis and promoted cell migration, providing a novel, optimized strategy for improving the therapeutic efficacy of BMSCs transplantation for ischemia [218]. Beyond used as therapeutic drugs, exosomes can also serve as delivery vehicles for therapeutic macromolecules in NBDD, and the present studies include hypoxia‐specific anti‐RAGE exosomes carrying anti‐miR‐181a oligonucleotides in an IS model, and BDNF‐loaded small EVs for CI therapy, which can selectively target the peri‐infarct region [77, 239]. In light of the significant therapeutic potential of nucleic acid therapy in stroke, microRNA [226], siRNA [223], recombinant human nicotinamide mononucleotide adenylyltransferase 1 [224], and IN delivery of nucleotides [225] can reduce brain infarction, decrease BBB permeability, and mitigate cellular apoptosis in brain ischemia. On the other hand, melatonin [227, 228], guanosine [229, 230], apelin‐13 [233], the neuroprotective peptide NR2B9c [231], and Fas‐blocking peptide [232] have been developed for IN delivery as promising neuroprotective therapies for ischemic injuries, which can suppress oxidative stress, inflammation, apoptosis and increase angiogenesis. Among these, NPs carrying the neuroprotective peptide NR2B9c can bypass the BBB, delivering NR2B9c to the brain and neurons, thereby protecting them from excitotoxicity [231]. Recently, it has also been found that IN administration insulin and IGF‐1 promoted survival of IS animals [237, 301, 302]. Similarly, the study also highlights that NGF [238], human IL‐1RA [240], IL‐1b antagonist [241], oxytocin [242], bFGF [244], acidic fibroblast growth factor (aFGF) [245], recombinant human erythropoietin [246, 247, 248], dynorphin A (1–8) [249, 250], and exendin‐4 [243] have been proven to be promising drugs for delivery from nose to brain to treatment stroke. Most notably, IN delivery of a caspase‐1 inhibitor in the treatment of global CI (GCI) targets ischemic neuronal injury and functional deficits following transient GCI [234]. Multiple other studies demonstrated that Wnt3a reduces neuronal apoptosis and improves neurological recovery after MCAO in rats via the Frizzled‐1/PIWI1a/FOXM1 pathway [256]. It also alleviates neuroinflammation by modulating the responses of microglia and astrocytes, positioning IN Wnt3a as a promising strategy for neuroprotection and regeneration following IS [257, 258]. As another study, NBDD of gelatin NPs enhances the neuroprotective effects of osteopontin (OPN) in IS, while the OPN heptamer peptide (OPNpt7R) with the RGD motif also provides neuroprotection by reducing inflammation, suppressing M1 microglia markers, and promoting M2 polarization [254, 255]. Furthermore, it has been discovered that mitochondria and β‐1, 3‐galactosyltransferase 2, which are potential drugs through NBDD, both offer neuroprotective effects in IS by reducing oxidative damage and inflammation [252, 253].

Similarly, they found that herbal components are beneficial to treat various diseases via multiple targets and IN delivery has the potential to engage with a variety of herbal components, such as salvinorin A [260, 261], baicalin [259], ginsenoside Rg3 (Rg3) [263], thymoquinone [262], cimicifugoside H‐1 [266], resveratrol [267], scutellarin [268], breviscapine [269], rutin [270], salvianolic acid B [271], eugenol [272], and catalpol [265] for use in stroke treatment. In a rhesus monkey IS model, IN salvinorin A reduces infarct volume and improves neurological outcomes [261]. Multidrug (baicalin, borneol, and cholic acid)‐loaded liposomes, combined with IN administration, increase brain‐targeted accumulation [259]. Furthermore, recent discoveries have highlighted that macrophage membrane‐cloaked liposome–Rg3/PNS, a promising biomimetic nano‐drug delivery system, can effectively bypass mononuclear phagocytosis, target inflammatory endothelial cells and microglia, traverse the BBB, and ultimately achieve targeted delivery to ischemic sites [263]. Catalpol, when delivered through IN, has been shown to significantly reduce brain cell apoptosis, enhance Bcl‐2 expression, inhibit Bax expression, and mitigate oxidative stress by upregulating Nrf2 and HO‐1 [265]. Another study demonstrated that rutin‐encapsulated chitosan NPs could effectively target the brain [270]. Collectively, these studies underscore the potential of IN delivery for stroke treatment using a variety of drugs. However, while IN administered drugs have demonstrated therapeutic feasibility in animal models of stroke, there is a paucity of clinical research in this area, warranting further exploration.

#### 3.2.2 Traumatic Brain Injury

TBI is the third leading cause of death worldwide. It results from one or more impacts involving severe mechanical forces to the brain, causing various neurological changes [303]. In recent years, drug‐based IN delivery therapies have been investigated in the course of TBI treatment.

Recent studies indicate the promising potential of IN treatments for TBI using various agents, including lidocaine [273], ALM (a combination of adenosine, lidocaine, and magnesium) [275], the SUR1 inhibitor glibenclamide [274], and tissue plasminogen activator (tPA) [276]. In a cohort of 15 patients experiencing spasticity following TBI over a 1‐year period, IN administration of 0.5% lidocaine demonstrated efficacy in reducing muscle spasms [273]. Furthermore, researchers have observed that ALM therapy does not induce toxic or inflammatory effects on human nasal epithelial cells, thereby facilitating future in vivo toxicity studies and potential clinical applications for TBI treatment [275]. Meng et al. [276] reported that subacute IN administration of tPA enhanced functional recovery, promoted neurogenesis in the brain, and stimulated axonal sprouting in the spinal cord post‐TBI.

Beyond pharmacological agents, IN delivery also holds promise for the administration of stem cells, stem cell‐derived EVs, and proteins in TBI treatment. The IN delivery of stem cells and EVs is particularly noteworthy, as it provides neuroprotection, functional neural regulation, and neurotrophic support during the recovery phase. For example, the administration of L‐myc‐expressing human neural stem cells via NBDD has demonstrated migration along white matter tracts, with projections toward the hippocampus and regions impacted by TBI [277]. A single IN dose of hMSC‐EVs administered 90 min post‐TBI has been shown to mitigate TBI‐induced reductions in BDNF–ERK–CREB signaling, hippocampal neurogenesis, and synapse formation [278]. Furthermore, IN delivery of hASC‐EVs 48 h postinjury has been found to alleviate motor and cognitive impairments following TBI [279]. EVs derived from human pluripotent stem cell‐derived glial cells also utilize this delivery method to exert neuroprotective effects through the modulation of brain microRNA in TBI. [280] Additionally, PEV preparation isolated from platelet concentrate supernatant significantly showed anti‐inflammatory effects in a TBI mouse [281].

Apart from that, various proteins, including insulin, NGF, oxytocin [287], progesterone [304], human platelet lysate [289], agomir‐let‐7i [288], antisecretory peptide AF‐16 [291], KAFAK [290], wnt3a [293], OPN [294], and IL‐4 [295], have been developed for NBDD. These compounds hold potential for enhancing neuroprotective effects and promoting recovery of brain function following TBI. NGF has been shown to mitigate neuroinflammation, tau hyperphosphorylation, and protein aggregation, while also modulating various mitochondrial functions [282, 305]. Studies have demonstrated that acute IN administration of NGF reduces reactive astrogliosis, microglial activation, and IL‐1β levels in young rat models of TBI [283]. In clinical case reports, a 4‐year‐old boy [306] and a 14‐year‐old boy [307] with severe TBI were treated with IN NGF and human recombinant NGF, respectively. This treatment elevated NGF and doublecortin levels in the cerebrospinal fluid, leading to clinical improvements without reported side effects. Additionally, IN administration of insulin has been found to enhance cerebral glucose uptake, reduce neuroinflammation and hippocampal lesion volume [284, 285]. Notably, insulin can be coadministered intranasally with hypothermia [286]. By way of the NBDD, human platelet lysate and Agomir‐let‐7i significantly improve cognitive function and suppress neuroinflammation, glial scar formation, and neuronal apoptosis in TBI mice [288, 289].

Herbal components are crucial factors that promote blood circulation, remove blood stasis, have anti‐inflammatory effects, and improve microcirculation, all of which can help alleviate secondary damage after TBI. For instance, the IN administration of paclitaxel significantly imparts neuroprotection against brain injury and cognitive impairment in mice [297]. It is puzzling that the Icariin/HP‐β‐cyclodextrin inclusion complex via facial intradermal administration preserved more neurons and oligodendrocytes, and decreased microglia and astrocyte activation compared with IN administration [308]. This result emphasize that IN administration is not the best way for some drugs to exert its therapeutic effect in CNS disease. In addition, it appears that curcumin and resveratrol which has a preventive effect in inhibiting neuroinflammation and cognitive decline and has been determined to have a protective effect on TBI. These results emphasize the potential of curcumin and resveratrol as promising therapeutic drugs by NBDD to treatment TBI [309].

What is more, a novel therapeutic approach can be devised by concentrating on NBDD of herbal components for TBI. Despite IN administration has shown neuroprotective effects on acute neurological disease in animal experiments, such as reducing brain damage, lowering inflammatory responses, and alleviating oxidative stress, current research is still relatively limited. In this regard, future research can further optimize the dosing regimen, explore the NBDD effects of different drugs, and conduct clinical trials to verify their safety and efficacy.

### 3.3 Brain Tumor

#### 3.3.1 Glioblastoma

Brain cancer is a serious condition caused by the growth of abnormal cells in the brain. Gliomas, which make up about 80% of all malignant primary brain tumors, are among the most common. Glioblastoma (GBM) is a type of astrocytic tumor, having the highest mortality rate among all gliomas [310]. Traditionally, the therapeutic approach for GBM involves maximal surgical resection, followed by adjuvant chemotherapy and radiation therapy. Recent research efforts are promisingly directed toward improving the delivery of chemotherapeutic agents to the brain through NBDD, addressing the unmet therapeutic needs in the treatment of brain‐related disorders [73] (Table 4).

**TABLE 4:** Summary of the application and function of intranasal drug delivery in brain tumor.

Diseases	Drug	Animal model in vivo/in vitro	Intranasal condition	Mechanism of action	Nose‐to‐brain drug delivery system	References
GBM	Carmustine	A172 cell line; U87 MG cell line; nasal tissue of the goat	5.707 mg/kg; 0.81 mg/kg 20 µL	/	NPs; chitosan coated NPs; thermoreversible mucoadhesive in situ intranasal gel	[311, 312, 313]
	Simvastatin	U‐138 MG cell line and C6 cell line	60 µg; 30 µL/nostril	Decrease in tumor growth and malignancy	Chitosan‐coated lipid‐core nanocapsules	[314]
	Teriflunomide	U‐87MG cell lines; sheep nasal mucosa	l1 mg/kg; 2 mg/kg; 4 mg/kg	/	Mucoadhesive microemulsion	[315]
	Sunitinib	Rat C6 glioma cell	15 mg/kg/day	Reduction in tumor growth; inhibition of angiogenesis	/	[316]
	Doxorubicin	Rat glioblastoma C6 cell lines	0.35 mg/kg; 25 µL	Induction of apoptosis	PLGA NPs	[317]
	Mebendazole	Orthotopic C6 rat model	/	/	Microemulsion	[318]
	Bevacizumab	Orthotopic C6 rat model; orthotopic GBM nude mice model	5 mg/kg	Reduction in tumor growth; higher antiangiogenic effect	In situ gel loaded mesoporous silica NPs; polymeric NPs	[319, 320]
	Ribavirin	/	/	/	In situ system	[321]
	Gemcitabine	U215 and T98G cell lines	/	/	Chitosan‐PLGA mucoadhesive NPs	[322]
	Disulfiram	C6 and U87 cells; glioma‐bearing rats	10 mg/kg	Inhibition of tumor growth	Ion‐sensitive nanoemulsion in situ gel	[323]
	Temozolomide	C6 rat model; 16HBE cell lines and T98G cells lines; U‐87MG and L929 cell lines; porcine nasal mucosa	0.3 mg/kg/day, 50 µL	/	Conjugated Gold NPs; NPs; nanoemulsion; lipid nanovesicles	[324, 325, 326, 327, 328, 329]
	Ponatinib	/	/	/	Molecular‐gated mesoporous NPs	[330]
	LOM and PG	Rabbit nasal mucosa; U251 glioblastoma cell line	/	/	Liposomes	[331]
	Methotrexate	Rat nasal mucosa	1.25 mg/kg	/	Thermosensitive PLA based nanodispersion	[332]
	miR17	Glioblastoma cell line GL‐26	20 nmol/2 µL; 20 µL	Inhibits GL26 tumor growth	Grapefruit‐derived nanovector	[333]
	c‐Myc siRNA	U87 MG cells, brain capillary endothelial cells bEnd.3; epithelial cells Calu‐3; rrthotopic glioma model	0.67 mg/kg	Reduction of apoptosis	Lipoplex with a core–shell structure	[334]
	Anti‐Gal‐1 siRNA	Murine GL261 glioma cells and human primary culture glioblastoma cells	/	Decreased expression of Gal‐1; reduction of myeloid suppressor cells and regulatory T cells	Chitosan NPs	[335, 336]
	siRNA	In situ GL261 glioma model	0.25 mg/kg	Promotion of DC maturation through the NF‐κB and Erk1/2 MAPK pathway	Self‐assembling siRNA delivery system based on brain tumor‐targeted T7 peptide	[337]
	AntimiRNA‐21 and miRNA‐100	Orthotopic GBM mouse models; U87MG and GL26 glioma cells	/	Prominent tumor regression	CXCR4‐engineered microfluidically processed EVs; Polyfunctional gold–iron oxide NPs	[338, 339]
	MSCs	U87MG glioma cell line	5 × 10^5^ of MSCs; 3 µL	Killing of glioma cells in vitro	/	[340]
	NSCs	U87MG cells; patient‐derived xenograft mouse model	5 × 10^5^, 12 µL	/	/	[341]
	RAP and antagomir‐21	C6 glioblastoma cells; intracranial tumor rat model	/	Decreased levels of vascular endothelial growth factor and miR‐21	Self‐assembled NPs	[342]
	Melatonin	U87MG glioblastoma cells and MRC‐5 nontumor cells	/	/	Polycaprolactone NPs	[343]
	Camptothecin	Glioma model rats	/	/	Tat‐modified nanomicells	[344]
	Luteolin	SH‐SY5Y neuroblastoma cells; Fresh porcine nasal membranes	/	/	Chitosan‐coated nanoemulsion	[345]
	Gastrodin	GL261 mouse glioblastoma cell lines	/	/	Borneol‐modified and gastrodin‐loaded liposome	[346]
	Temozolomide and resveratrol	LN229 cell lines	/	/	Nanostructured lipid carriers	[347]
	Kaempferol	Freshly isolated porcine nasal mucosa; C6 cell line	1.0 mg/mL; 50 µL	Induction of apoptosis	Mucoadhesive nanoemulsion	[348]
	Baicalin	U87 cells	10 mg/mL; 100 µL	Induction of cell cycle arrest at S and G2/M phases; upregulation of P21 gene and decline in Nrf‐2, HO‐1, and VEGF gene expression	Lipid nanocapsules	[349]
	Thymoquinone	U87 MG	1.5 µg/mL 100 µL	/	Self‐assembled lipidic nanovesicles; lipo‐polymeric nanoshells	[350, 351]
	Perillyl alcohol	/	54 µL	/	/	[352]
	Paclitaxel	U87 human glioblastoma cell line	5 mg/kg	/	NPs; arginyl–glycyl–aspartic tripeptide conjugated paclitaxel‐loaded NPs	[353, 354]

Abbreviations: DC, dendritic cell; Erk1/2, extracellular signal‐regulated kinase 1/2; GBM, glioblastoma multiforme; HO‐1, heme oxygenase‐1; LOM, lomustine; MAPK, mitogen‐activated protein kinase; NF‐κB, nuclear factor‐kappa B; Nrf‐2, nuclear factor erythroid 2‐related factor 2; NSCs, neural stem cells; PG, n‐propyl gallate; RAP, RAGE‐antagonist peptide; U87 MG, human glioblastoma cell line U87 MG; VEGF, vascular endothelial growth factor.

The most widely used methods for delivery via NBDD include NPs, microemulsions, in situ gels, and liposomes, which load different drugs such as carmustine [311], simvastatin [314], teriflunomide [315], mebendazole [318], ribavirin [321], gemcitabine [322], and disulfiram [323]. These approaches not only enhance the therapeutic efficacy by specifically targeting tumor sites but also significantly mitigate the toxicity to normal tissues, thereby presenting a promising novel strategy for GBM treatment. For example, researchers have designed molecular‐gated mesoporous NPs to increase ponatinib delivery to the brain—nanocarriers targeting specific cells or molecules can open under specific pH conditions, allowing them to remain in the body for a long time and continuously release drugs [330]. Moreover, IN delivery of cancer‐targeting doxorubicin‐loaded PLGA NPs induces apoptosis in tumor cells while sparing normal brain cells [317]. Similarly, the application of sunitinib via NBDD represents an innovative therapeutic approach that inhibits angiogenesis in GBM with reduced hepatotoxicity, and it is proposed as a safe method for GBM treatment [316]. A recent study highlighted the potential of a liposomal formulation combining lomustine and n‐propyl gallate for targeting GBM via the IN route [331]. Notably, the IN delivery of temozolomide from lipid nanovesicles, which are surface‐decorated with transferrin and coencapsulated with miltefosine, facilitates the drug's penetration into the brain by bypassing the BBB in glioma‐bearing mice. This approach ensures efficient payload release upon reaching the brain, demonstrating that targeted drug synergy holds promise for delivering high therapeutic efficacy and could serve as a foundation for future clinical applications [324].

In recent years, targeted therapy and IN nucleic acid therapy, which focus on specific gene mutations or tumor microenvironments, have emerged as novel research avenues for treating GBM. IN siRNA delivery plays a pivotal role in the rapid transport to the brain and targeting of tumor cells, including miR‐17 [333], siRNA [337], c‐Myc‐targeting siRNA [334], anti‐Gal‐1 siRNA [335, 336], and therapeutic miRNAs such as miR‐100 and anti‐miR‐21 [338]. The self‐assembling siRNA NBDD system can reach the tumor site through the olfactory pathway, reducing drug loss and converting the “cold” tumor microenvironment to a “hot” tumor microenvironment [337]. Studies have shown significant alterations in the tumor microenvironment with IN delivery of siRNA targeting Gal‐1 loaded in chitosan NPs, which protected it from RNase degradation, reduced myeloid‐derived suppressor cells and regulatory T cells, and increased CD4+ and CD8+ T cells [335, 336]. Therapeutic miRNAs (miR‐100 and anti‐miR‐21) IN delivery via gold‐iron oxide NPs and CXCR4‐engineered mpEVs, successfully bypassed the BBB, thereby improving the overall survival rate of mice and providing new ideas for clinical treatment [338, 339]. Moreover, an antisense microRNA oligonucleotide against miR‐21 also was suggested as an IN therapeutic nucleic acid for GBM [355]. Similarly, stem cell‐based therapeutics such as MSCs and NSCs delivered via IN are an effective treatment approach, enabling repeated use of modified stem cells to target malignant glioma [340, 341]. MSCs serve not only as an IN therapeutic agent but also as a delivery vector to carry sorafenib to GBM, reducing angiogenesis [340, 356]. However, this approach could limit the action of the released sorafenib. A growing body of evidence shows that other drugs like methotrexate [332], melatonin‐loaded polycaprolactone NPs [343], RAGE‐antagonist peptide, and antagomir‐21 [342] have been delivered by NBDD for the treatment of GBM, showing strong activity not only without toxicity to nontumor cells but also with effects on reducing angiogenesis in tumors.

Another significant category of antitumor agents consists of compounds derived from natural sources. Compounds such as camptothecin [344], luteolin [345], gastrodin [346], resveratrol [347], kaempferol [348], baicalin [349], and thymoquinone [350, 351] have demonstrated substantial efficacy in the treatment of GBM through NBDD. Specifically, luteolin‐loaded chitosan‐coated nanoemulsions have been shown to inhibit the growth of neuroblastoma cells at elevated concentrations [345]. In addition, a novel therapeutic approach involving borneol‐modified and gastrodin‐loaded liposomes has been found to not only enhance the efficacy of temozolomide in treating GBM but also to disrupt the communication between astrocytes and GBM cells, reduce the expression of the Cx43 protein, and extend the survival of GL261 tumor‐bearing mice [346]. Nonetheless, the combination index of temozolomide and resveratrol in nasal cavity shows synergistic anticancer activity at different ratios [347]. Notably, perillyl alcohol (POH), a naturally occurring monoterpene, warrants particular attention. Its IN administration leads to a ten‐fold increase in the CSF‐to‐plasma ratio of POH [352]. Therefore, a phase I/II study showed that daily IN administration of 440 mg POH improved overall survival in patients with recurrent GBM [357]. Another phase I trial showed that IN NEO100 (highly purified perilla alcohol) has good treatment tolerance in adult patients with recurrent GBM and is associated with improved survival rates [358]. A recent study has found that in rodent models of GBM, IN codelivery with NEO100 demonstrates effective brain targeting and enhances the therapeutic impact of bortezomib [359]. Other research has shown that the NP‐based delivery of cancer‐targeting paclitaxel, specifically utilizing NBDD, amplifies antitumor effects in malignant GBM [353]. The codelivery of paclitaxel and miltefosine by nanovesicles or lipid NPs has a synergistic effect and can improve the effectiveness of GBM treatment while reducing toxicity [360, 361]. Additionally, recent in vitro studies have revealed that curcumin can effectively modulate the polarity of microglia, thereby inhibiting the proliferation and dissemination of GBM. However, further investigation is necessary to assess its potential for IN delivery [362].

Preliminary investigations indicate that IN administration may substantially enhance drug concentrations within the brain and effectively target tumor cells. Research on IN administration for the treatment of GBM remains predominantly in the preclinical or early clinical trial phases. Nonetheless, additional clinical validation is required to ascertain the efficacy and safety of IN administration in practical therapeutic contexts, as well as to elucidate the functional mechanisms underlying drug delivery through this route and its potential to augment therapeutic efficacy.

### 3.4 Psychiatric Disorders

Psychiatric disorders are complex syndromes characterized by intricate neurological underpinnings. These disorders include depression, anxiety, autism spectrum disorder (ASD), attention deficit hyperactivity disorder, and schizophrenia [363]. Depression is the most prevalent and disabling, characterized by high incidence and low remission rates. Depression's pathophysiology involves changes in several biological pathways, such as hypothalamic–pituitary–adrenal axis hyperactivity, imbalances in the oxidant/antioxidant system, increased inflammatory cytokines, reduced BDNF levels, and diminished serotonergic neurotransmission in different brain areas [364]. The complexity and patient heterogeneity in depression's pathogenesis present substantial challenges to pharmacotherapy (Table 5).

**TABLE 5:** Summary of the application and function of intranasal drug delivery in psychiatric disorders.

Diseases	Drug	Animal model in vivo/in vitro	Intranasal condition	Mechanism of action	Nose‐to‐brain drug delivery system	References
Depression	Escitalopram	LPS model	228 µL/animal (containing 2.38 mg/kg)	Reduction of NF‐κB and TNF‐α expression	Nanostructured lipid carrier thermosensitive gel	[365]
	Agomelatine	CUMS model	2.6 mg/kg	Increased levels of BDNF; Reduction of proinflammatory cytokine levels	Thermosensitive hydrogel; mucoadhesive‐nanoemulsion‐gel system; solid lipid NPs; mucoadhesive thermosensitive in situ gel; microemulsion	[366, 367, 368, 369, 370]
	Venlafaxine	/	/	/	Hyaluronic acid‐coated transbilosomes; novasomes; nanostructured lipid carrier; chitosan NPs; alginate NPs	[371, 372, 373, 374, 375]
	Desvenlafaxine Succinate	/	/	/	Bilosomes in thermosensitive hydrogel	[376]
	Desvenlafaxine	Stress‐induced model	5 mg/kg/day	/	PLGA–chitosan NPs	[377]
	Duloxetine	/	0.54 mg/day	/	Lipid nanocarriers in situ cubo‐gel; thiomer gel	[378, 379, 380]
	Sertraline	CUMS model	4.87 mg/kg	Reduction of adrenaline; Increase in serotonin and melatonin levels.	/	[381]
	Paroxetine	Chronic depression‐induced rats	0.704 mg/kg	Decrease in elevated TBARS levels	Nanoemulsion; chitosan‐modified polymeric NPs	[382, 383]
	Dantrolene	5XFAD and wild‐type B6SJLF1/J mice	5 mg/kg	Inhibition of pathologically increased expression of RyR‐2 and InsP3R‐1, MDA‐modified proteins, pyroptosis regulatory proteins, and neurotoxic cytokines; increase in IL‐10 and synaptic proteins	NPs	[384]
	Fluoxetine and embelin	CUMS model	1.8 mg/kg for 8 weeks	Reduction of IL‐6 and TNF‐α levels; decrease in SOD and CAT levels	Nanoliposomal in situ gel	[385]
	D1–D2 interfering peptide	/	Doses of >1.67 nmol/g (5.75 mg/kg); 8–12 µL, 50 mM	/	/	[386]
	IGF‐1	/	150 µg; 50 µL	/	/	[387, 388]
	GLP‐1 analog lixisenatide	CUMS model	50 mg/kg/d, 50 µL	Increased BrdU+ cells and BrdU+/DCX+ cells; improvement of adult neurogenesis and related behaviors; increased phosphorylation of CREB protein	/	[389]
	GLP‐2	A549 cell line	60 µg/kg, 3.6 µg/mouse	/	Nasal formulation of GLP‐2 containing 5% polyoxyethylene (25) lauryl ether and 1% β‐cyclodextrin; GLP‐2 derivative containing cell penetrating peptides and a penetration accelerating sequence	[390, 391, 392]
	BDNF	CMS model; MCAO combined with isolation and CUMS to model PSD; CUMS model	10 µL (about 8.32 × 10^7^ genome copies of AAV vector); 20 µL; 40 µg/kg	Enhancement of hippocampal BDNF content; reduced IL‐6, TNF‐α, iNOS and IL‐1β expressions; inhibition of oxidative stress	Recombinant AAV containing BDNF fused with HA2TAT	[393, 394, 395]
	Resolvin E1	LPS and PSL‐induced depression model	10 ng/mouse	/	/	[396]
	NGF	CUMS model	75 µg/kg, 2.5 µL; 50 µg/kg, 10 µL	Increased levels of norepinephrine and dopamine; effect on the number of 5‐BrdU, c‐fos, and caspase‐3 positive neurons	/	[397]
	Monophosphoryl lipid A	CUS model	10/20 µg/mouse	Stimulation of microglia	/	[398]
	M2‐SF	/	30 µL	Down‐regulation of proinflammatory cytokines	/	[399]
	IL‐4	CUMS model	1 ng/mouse	Restoration of NRF2, NF‐κB, IL‐1β, IL‐4, BDNF, and IDO expression; modulation of oxidative stress markers	/	[400]
	circATF7IP siRNA	LPS‐induced depression‐like model	0.1 mg/kg; 0.3 mg/kg; 1 mg/kg	Reduction in proinflammatory cytokine production	Lipid NPs	[401]
	Albiflorin	CUMS model	0.35 mg/kg	Decreased levels of proinflammatory cytokines, and repaired neuronal damage	Thermosensitive hydrogel	[402]
	Icariin	CUMS model	20 mg/kg	Reversal of testosterone, IL‐6, and PGE2 levels in the plasma	Nanogel‐thermoresponsive hydrogel system	[403]
	Genipin	Reserpine‐induced mouse model	6 mg/kg	Improvement of abnormal levels of neurotransmitters	Thermosensitive hydrogel	[404]
	Resveratrol	CUMS model	44.1, 88.2, 176.4 µg/kg	Increase in the concentration of 5‐HT, DA, NE, and 5‐HIAA	Thermosensitive hydrogel	[405]
	Berberine	CUMS and reserpine‐induced model	0.05, 0.10, 0.15 mg/kg	Restoration of mitochondrial dysfunction, phospholipid, and sphingolipid abnormalities	In situ thermoresponsive hydrogels	[406]
	Berberine and evodiamine	Reserpine‐induced rat model	BBR: 0.05 mg/kg; EVO: 0.008 mg/kg	Regulation of abnormal levels of monoamine neurotransmitter metabolism and related metabolic pathways	Self‐assembled thermosensitive in situ hydrogels	[407]
	Quercetin	FST model	10 mg/kg	Lowest levels of c‐fos protein expression	Nanotransferosomal gel	[408]
	White tea	CUMS model	20 mg/kg; 40 mg/kg	Enhanced expression of BDNF and OMP	/	[409]
	Esculin hydrate	/	50 mg/kg, 50 µL	/	Surface‐engineered niosomes	[410]
PTSD	NPY	PTSD model	150 µg	Reduction of depressive‐like behavior	/	[411]
Anxiety	shRNA	/	40 µL	/	AAV9 vectors	[412]
ASD	Dopamine	Genetic ASD model	3 mg/kg	Enhancement of TH levels	/	[413]
	hUC‐MSCs‐EVs	/	/	/	/	[414]
	Nasal Olfactory Mucosal MSCs‐EVs	Murine model induced by MAM	1.4 × 10^11^	Reduction in neuroinflammatory markers and suppression of microglial activation; upregulation of PSD95 and TH	/	[415]

Abbreviations: 5‐HIAA, 5‐hydroxyindoleacetic acid;5‐HT, serotonin; ASD, autism spectrum disorder; BBR, berberine; BDNF, brain‐derived neurotrophic factor; CAT, catalase; CMS, chronic mild stress; CREB, cAMP response element‐binding protein; CUMS, chronic unpredictable mild stress; DA, dopamine; EVO, evodiamine; FST, forced swimming test; GSH, glutathione; HC, hippocampus; IDO, indoleamine 2,3‐dioxygenase; IFNγ, interferon‐gamma; IL, interleukin; InsP3R‐1, inositol 1,4,5‐trisphosphate receptor type 1; LPS, lipopolysaccharide; M2‐SF, M2 macrophage supernatant; MAM, methylazoxymethanol; MCAO, middle cerebral artery occlusion; MDA, malondialdehyde; NE, norepinephrine; NF‐κB, nuclear factor‐kappa B; NGF, nerve growth factor; NLRP3, NLR family pyrin domain containing 3; NPY, neuropeptide Y; NRF2, nuclear factor erythroid 2‐related factor 2; OMP, olfactory marker protein; PFC, prefrontal cortex; PGE2, prostaglandin E2; PTSD, posttraumatic stress disorder; RyR‐2, ryanodine receptor type 2; SOD, superoxide dismutase; TBARS, thiobarbituric acid reactive substances; TNF‐α, tumor necrosis factor‐alpha.

Notably, NBDD utilizes first‐line antidepressants, peptides, natural active ingredients, and other substances with antidepressant properties. The US FDA approved IN esketamine, the S‐enantiomer of ketamine, for treating major depressive disorder (MDD) in adults [19]. Recently, escitalopram [365], agomelatine [366], venlafaxine [371], duloxetine [378], sertraline [381], paroxetine [382], desvenlafaxine [377], and other antidepressants were considered as IN drugs in the chronic unpredictable mild stress (CUMS) model. The results showed that by loading onto carriers such as a mucoadhesive nanoemulsion gel system, translosomes, thermometers, NPs, and nanostructured lipid carriers, these drugs can target the brain and avoid first‐pass metabolism in the liver, improving their bioavailability and thereby enhancing their antidepressant effects [367, 372, 373, 374, 379, 383]. It is worth mentioning that histopathological studies of the nasal epithelium have shown no damage or inflammation during treatment. IN coadministration is known to have synergistic potential and reduce adverse reactions compared with administration alone. For example, Escitalopram and Paroxetine are encapsulated in nanostructured lipid carriers, nanoliposomal in situ gel of fluoxetine and embelin were formulated as IN coadministration systems, which can reduce exposure to the whole body and lungs, improve antioxidant enzyme levels, and reduce inflammatory markers in the CUMS depression animal model [385, 416].

Owing to their unique characteristics in therapy, many protein and peptide drugs are IN administered, which can lead to a direct transfer of drugs from the nose to the brain in similar or higher concentrations than those obtained through systemic administration. For example, D1–D2 interfering peptide [386], IGF‐1 [387], GLP‐1 analog lixisenatide [389], GLP‐2 [390, 391], BDNF [393, 394], and NGF [397] were considered effectively IN delivered to the CNS in both animals and humans. Researchers using the pressurized olfactory device, a novel IN delivery system, deliver the D1–D2 interfering peptide, and an antidepressant‐like effect can be detected within 2 h [386]. To enhance BDNF delivery to the CNS, it was fused with cell‐penetrating peptides (TAT and HA2) and packaged in an adeno‐associated virus to construct the BDNF–HA2TAT/AAV for IN delivery, which increases hippocampal BDNF and exerts antidepressant effects in CMS mice, indicating that this strategy has promising therapeutic potential for MDD [393, 394]. Another example is resolvin E1, a bioactive lipid mediator derived from eicosapentaenoic acid for IN administration, which produces antidepressant‐like effects through activity‐dependent BDNF/VEGF release as well as mTORC1 activation [396]. It is worth mentioning that NBDD of IL‐4 and M2 macrophage‐derived soluble factors, which can modulate neuroinflammation and oxidative stress, exert antidepressant‐like effects as an innovative strategy for depression treatment [399, 400]. In an open trial, patients who had not responded to 40 mg of escitalopram were administered IN synthetic oxytocin over a period of 4 weeks, and the results showed that patient's scores on the Hamilton Depression Rating Scale were significantly reduced [417].

Additionally, many herbal components have been successfully formulated in NBDD systems for brain targeting. For example, albiflorin [402] and Icariin [403], which are drugs used to decrease levels of proinflammatory cytokines and repair neuronal damage in CUMS rats, have been formulated in a nanogel that improves its antidepressant effects through IN administration. The in vivo study of the developed THS loaded with genipin [404], resveratrol [405], or berberine [406] showed higher brain targeting, greater bioavailability in the brain, and increased concentrations of neurotransmitters in brain tissue due to the unique formulations and administration methods. Moreover, the IN codelivery of berberine and evodiamine by self‐assembled thermosensitive hydrogels, showed good release properties and achieved antidepressant effects by regulating monoamine neurotransmitter metabolism and related metabolic pathways, providing a noninvasive treatment strategy for the clinical treatment of depression [407]. Quercetin can be used as a crosslinker and therapeutic drug. When loaded with nanotransferosomal gel, it substantially alleviated depressive symptoms in rats [408]. Xu et al. [418] developed quercetin‐based alginate nanogels loaded with BDNF, using a thermosensitive gel for combination therapy of depression via IN delivery. These nanogels effectively bypass the BBB through the nose‐to‐brain pathway and protect BDNF from oxidative damage, offering a promising approach for treating depressive disorders through IN administration [418].

There are many drugs for anxiety that were reported such as NPY [411], shRNA [412], and quercetin [419]. IN delivery of AAV9 vectors carrying shRNA led to reduced anxiety‐like behavior in mice and a notable enhancement in memory, with improvements of 104% in mice and 92% in rats, compared with vehicle‐treated controls [412]. ASD is a prevalent neurodevelopmental condition. DA emerges as a compelling candidate for study, with findings indicating that DA alterations manifest differently across contexts. Notably, DA treatment ameliorates behavioral deficits in both mouse models, suggesting its potential as a therapeutic avenue for ASD [413]. Recent advancements in stem cell therapy have generated optimism for treating mental disorders. hUC‐MSCs‐EVs, which effectively penetrate brain tissue via the IN route, have been shown to restore social behaviors and correct repetitive stereotypical actions in mice [414]. Concurrently, IN administration of nasal olfactory mucosal MSCs‐EVs resulted in a reduction of neuroinflammatory markers and suppression of microglial activation in the hippocampus. This approach offers a multifaceted strategy to address the mechanisms underlying schizophrenia and holds promise for innovative treatments of this complex disorder [415].

Some antidepressant active ingredients, such as peptides, natural active ingredients were used by NBDD, which have demonstrated that they are promising therapeutics for psychiatric disorders. However, most research is limited to cells and animal models. Clinical trials are necessary to confirm safety and effectiveness. The development of new drug delivery methods for IN administration, such as protein‐targeted carriers, is also one of the main directions of exploration to improve the efficacy of antidepressant treatment in the future.

## 4 Challenges and Limitations

IN administration offers an effective approach to address the intricate pathophysiological challenges of brain diseases and enhance drug delivery to the brain. The delivery of drugs from the nose to the brain is influenced by the nose's physiological characteristics, the physicochemical properties of drug active ingredients, and the drug's dosage form.

### 4.1 Biological Barriers

The physiological structure of the nose inherently serves as a barrier, effectively inhibiting the infiltration of drugs into the brain. The TJ and protective mucous membrane of the olfactory and respiratory epithelium are natural biological barriers that act as selective filters, reducing drug diffusion and permeability [420, 421]. Although most lipophilic compounds are more permeable to the nasal mucosa, peptides, macromolecules, and small hydrophilic molecule compounds are generally less permeable. Permeation enhancers have been shown to improve the absorption of high molecular weight antidepressants by facilitating the production of hydrophilic pores, and increasing membrane fluidity and permeability of TJs [422]. Additionally, nasal mucosal cilia clearance poses a challenge for drug delivery from the nose to the brain. The olfactory and respiratory surfaces are lined with numerous cilia that act as a barrier against external particles entering the nasal cavity. Nasal drug administration typically results in clearance within 20–30 min, impacting drug retention and absorption [423]. Despite the lower enzyme presence in the nasal cavity, enzymes like the cytochrome P‐450 system, glutathione S‐transferase, and proteolytic enzymes can still influence antidepressant absorption [424].

### 4.2 Challenges of Physicochemical Properties of Drugs

Besides physiological factors, the physicochemical properties of drugs play a vital role in the effectiveness of IN drug delivery. Drugs need to be sufficiently soluble in the nasal mucosa's environment for effective absorption [425]. However, some drugs may have poor solubility in water or other solvents, which can limit their ability to be absorbed into the bloodstream through the nasal passages [426]. Moreover, larger molecules, such as proteins, peptides, and certain biologics, may struggle to pass through the nasal mucosa due to their size [427]. The nasal epithelium acts as a barrier, limiting the permeability of larger molecules. Some drugs may undergo chemical degradation, lose potency, or have their effectiveness reduced when exposed to these conditions [428]. The pH of a drug formulation is considered to affect its stability and the irritation it may cause in the nasal cavity. If the pH is too high or too low, it could damage the mucosa or cause discomfort [429]. Additionally, some drugs may not be stable or effective at certain pH levels.

### 4.3 Challenges of Clinical Translation

Although nasal drug delivery has many promising advantages, such as noninvasiveness, rapid onset, and avoidance of first‐pass metabolism, it faces significant challenges in clinical translation [430]. These challenges span drug absorption efficiency, device accuracy, patient compliance, and formulation stability. At present, research on IN delivery is mostly limited to in vivo animal models of rodents, and data from large animals are scarce for preclinical studies. Therefore, in addition to studying small animals, it is recommended to conduct preclinical studies on IN delivery of drugs or drug delivery devices in large animals such as rabbits and monkeys. Of course, IN delivery also face various challenges from preclinical to market. Due to individual differences in nasal anatomy and patient compliance, personalizing treatment strategies and optimizing drug delivery devices are challenging. With the increase in research, quantifying the exact amounts of drugs per unit therapeutically remains difficult, and finding a strategy that allows precise control of IN therapy content is also challenging for scientists. As advances in nanotechnology, drug delivery devices, and formulation science continue, many of these challenges are expected to be addressed, and nasal drug delivery could see broader clinical applications in the future.

## 5 Future Directions and Perspectives

IN drug delivery holds significant promise for noninvasive treatment options and is an evolving field. As research and technology progress, several future directions and perspectives for IN drug delivery can be envisioned.

The development of innovative nanocarriers, such as NPs, liposomes, micelles, dendrimers, and inorganic NPs, is poised to revolutionize nasal drug delivery. These nanomaterials can improve drug stability, bioavailability, and controlled release, while overcoming barriers like the nasal mucosal layer. By enabling targeted drug delivery to specific regions of the brain, nanotechnology offers promising solutions for treating neurological conditions. The development of other targeted drug delivery systems, utilizing receptors or biomarkers present in the nasal mucosa, will enable precise drug delivery to specific sites, such as the brain or respiratory system. This approach not only enhances therapeutic efficacy but also minimizes systemic side effects. Additionally, the application of stem cell‐based therapies and their EVs for IN delivery is rapidly emerging as a research hotspot, offering new possibilities for regenerative medicine and disease treatment.

The future of IN drug delivery will also see a significant focus on the development of novel therapeutic agents. Peptides, proteins, and RNA‐based therapeutics (e.g., mRNA and siRNA) are particularly promising due to their potential to treat a wide range of diseases, including viral infections, genetic disorders, and CNS conditions. For instance, IN delivery of insulin, growth hormones, and monoclonal antibodies offers a viable alternative to oral administration. Furthermore, the exploration of herbal medicine components for IN delivery presents an exciting avenue, as many natural compounds exhibit potential therapeutic effects for CNS diseases. By addressing current challenges and embracing new technologies, the potential for nasal drug delivery in modern healthcare is vast and could transform patient treatment paradigms.

## 6 Conclusion

In this review, we summarized the structure of the BBB in both physiological and pathological states, the mechanisms and pathways for NBDD, and the applications of IN delivery drugs, including proteins, peptides, genes, stem cells, small molecule drugs, herbal ingredients, and so on, in the treatment of different CNS diseases. In neurological diseases, including neurodegenerative diseases, acute neural injury, brain tumors, depression, and psychiatric disorders, IN delivery and their pivotal drugs can facilitate the direct transport of the drug through the olfactory and trigeminal nerve pathways, effectively attenuate oxidative stress, inhibit neuroinflammation, and suppress neuronal apoptosis. In addition, different drug delivery systems are addressed. Under BBB obstruction conditions, NBDD seems to be a noninvasive method that plays a wide role in CNS treatment. While NBDD shows promise as an effective therapeutic drug delivery platform for neurological diseases, there are pressing issues in both foundational research and clinical application of IN drugs. Numerous fundamental research challenges remain, including understanding the physiological characteristics of the nose and the physicochemical properties of drugs. In clinical applications, these challenges span drug absorption efficiency, device accuracy, patient compliance, and formulation stability. Overall, despite the obstacles limiting the clinical application of NBDD, there is optimism that these challenges can be surmounted through more comprehensive ongoing and planned investigations, thereby positioning NBDD as a promising therapeutic approach for neurological disorders and beyond, as mentioned in the article.

## Author Contributions

Yi Qiu and Shiyuan Huang reviewed the literatures and wrote the manuscript. Fang Yan and Xi Peng were responsible for discussing the manuscript and making critical revisions to the logic and grammar. Li Yang, Li Peng, and Tao Liu drafted and polished the figures and tables. Xi Peng and Guosong Zhang provided financial support. Yi Qiu and Shiyuan Huang contributed equally to the first author while Xi Peng and Fang Yan were equated to the corresponding author. All authors have read and agreed on the submission a publication of this manuscript.

## Conflicts of Interest

Author Li Yang is an employee of Sichuan Youngster Technology Co., Ltd., but has no potential relevant financial or non‐financial interests to disclose. The other authors have no conflicts of interest to declare.

## Ethics Statement

The authors have nothing to report.

## Data Availability

The authors have nothing to report.
